# Mourning and melancholia revisited: correspondences between principles of Freudian metapsychology and empirical findings in neuropsychiatry

**DOI:** 10.1186/1744-859X-7-9

**Published:** 2008-07-24

**Authors:** Robin L Carhart-Harris, Helen S Mayberg, Andrea L Malizia, David Nutt

**Affiliations:** 1 Psychopharmacology Unit, University of Bristol, Bristol, UK; 2 Emory University School of Medicine, Atlanta, GA 30322, USA

## Abstract

Freud began his career as a neurologist studying the anatomy and physiology of the nervous system, but it was his later work in psychology that would secure his place in history. This paper draws attention to consistencies between physiological processes identified by modern clinical research and psychological processes described by Freud, with a special emphasis on his famous paper on depression entitled 'Mourning and melancholia'. Inspired by neuroimaging findings in depression and deep brain stimulation for treatment resistant depression, some preliminary physiological correlates are proposed for a number of key psychoanalytic processes. Specifically, activation of the subgenual cingulate is discussed in relation to repression and the default mode network is discussed in relation to the ego. If these correlates are found to be reliable, this may have implications for the manner in which psychoanalysis is viewed by the wider psychological and psychiatric communities.

## Background

'When some new idea comes up in science, which is hailed at first as a discovery and is also as a rule disputed as such, objective research soon afterwards reveals that after all it was in fact no novelty' [1].

The intention of this paper is to draw attention to consistencies between Freudian metapsychology and recent findings in neuropsychiatry, especially those relating to depression. A case will be made that findings in neuroimaging and neurophysiology can provide a fresh context for some of the most fundamental theories of psychoanalysis. In his famous paper 'Mourning and melancholia', Freud carried out an elegant application of psychoanalytic theory to the illness of depression. It is the task of this paper to parallel the psychological processes described by Freud with the physiological processes identified by modern clinical research in order to furnish a more comprehensive understanding of the whole phenomenon.

Under the tutelage of Meynert, Freud began his career as neurologist studying the anatomy and physiology of the medulla. Inspired by a Helmholtzian tradition (1821–1894) and a 'psycho-physical parallelism' made fashionable by the likes of Hering (1838–1918), Sherrington (1857–1952) and Hughlings-Jackson (1835–1911), Freud began to consider more seriously how a science of movements of energy in the brain might account for psychological phenomena [2]. It has been argued that Freud never truly abandoned his physiological roots [3,4] and that his early flirtations with psycho-physical parallelism continued to haunt 'the whole series of [his] theoretical works to the very end' [4].

This paper will begin with an overview of some key concepts of Freudian metapsychology (libido, cathexis, object cathexis, the ego, the super ego, the id, the unconscious, the primary and secondary psychical process and repression) and an attempt will be made to hypothesise their physiological correlates. This will be followed by a summary of 'Mourning and melancholia' and an extensive look at relevant findings in neuropsychiatry. Of special interest are neuroimaging findings in depression and induced depressed mood, deep brain stimulation (DBS) of the subgenual cingulate (Brodmann area 25/Cg25) for the treatment of intractable depression, electrical stimulation of medial temporal regions, and regional atrophy and glial loss in the brains of patients suffering from major depression.

Before beginning, it is important to make a few brief comments on the principle of psycho-physical parallelism. Drawing connections between psychological and biological phenomena was an approach that Freud was both critical of:

'I shall carefully avoid the temptation to determine psychical locality in any anatomical fashion' [5].

'Every attempt to discover a localisation of mental processes...has miscarried completely. The same fate would await any theory that attempted to recognise the anatomical position of the system [consciousness] – as being in the cortex, and to localise the unconscious processes in the subcortical parts of the brain. There is a hiatus here which at present cannot be filled, nor is it one of the tasks of psychology to fill it. Our psychical topography has *for the present nothing to do with anatomy*' [6].

And receptive to:

'All our provisional ideas in psychology will presumably some day be based on an organic substructure' [7].

The ambiguity in Freud's position can be explained by his criticism of the modular or 'segregationist' [8] approach and preference for a more dynamic model [9]. Essentially, Freud was opposed to 'flag polling' the anatomical causes of psychological phenomena but not the drawing of parallels between psychological and physiological processes:

'It is probable that the chain of physiological events in the nervous system does not stand in a causal connection with the psychical events. The physiological events do not cease as soon as the psychical ones begin; on the contrary, the physiological chain continues. What happens in simply that, after a certain point in time, each (or some) of its links has a psychical phenomena corresponding to it. Accordingly, the psychical is a process parallel to the physiological – "a dependent concomitant"' [9].

Integrating psychoanalysis with modern neuroscience is a difficult and controversial endeavour. It should be made clear from the outset what we believe it is possible for this approach to achieve. Psychoanalysis can be viewed on two levels: a hermeneutic, interpretative or *meaning *based level; and a metapsychological, *mental process *based level. The hermeneutic level is inherently subjective. The question has often been raised whether it is possible to identify spatiotemporal coordinates of subjective meaning. This view was shared by Paul McLean in his seminal book 'The triune brain in evolution' [10]:

'Since the subjective brain is solely reliant on the derivation of immaterial information, it can never establish an immutable yardstick of its own...Information is information, not matter or energy' [10].

It would be incorrect to align this position with dualism. Psychophysical parallelism is a materialist approach that acknowledges that meaning arises through time between networks of communicative systems. It must be stated that the evidence cited in this paper cannot logically validate psychoanalysis on the hermeneutic level and neither does it provide evidence for the efficacy of psychoanalysis as a treatment modality (see [11] for a review). What we believe it can do, however, is bring together converging lines of enquiry in support of the Freudian topography of the mind. The findings cited below describe changes in physiological processes paralleling changes in psychological processes; however, the objective measures do not shed any light on the specific content or meaning held within these processes. Aside from interpretation, much of Freud's work was spent theorising about dynamic psychical processes; energies flowing into and out of mental provinces, energy invested, dammed up and discharged throughout the mind. It is this metapsychological level of psychoanalysis that we believe is most accessible to integration with modern neuroscience.

## An introduction to some key terms of Freudian metapsychology

### Libido

'Libido means in psycho-analysis in the first instance the force (thought of as quantitatively variable and measurable) of the sexual drives directed towards an object – "sexual" in the extended sense required by analytic theory' [12].

From its earliest recorded use [13] the term 'libido' was used to connote the principal energy of the nervous system. Freud differentiated 'libido' from a more general 'psychical energy':

'We have defined the concept of libido as a quantitatively variable force which could serve as a measure of processes and transformations occurring in the field of sexual excitation. We distinguish this libido in respect of its special origin from the energy which must be supposed to underlie the mental processes in general' [14].

Freud's extended use of the term 'sexual' brought him into conflict with Jung, who argued that the principal energy of the nervous system was not inherently sexual [15]. Arguably, the two perspectives are not irreconcilable. We may view Freud's 'libido' in connection with the motivational drive system (see The id below) and the withdrawal and investment of cerebral energy (see The ego below). Jung's 'psychical energy' can be viewed less specifically as cerebral energy in general.

### Cathexis

The German original 'Besetzung' literally translates as 'occupation', 'filling' or 'investment'. The neologism 'cathexis' was one that Freud was not especially fond of [16]. Freud first used the term on an explicitly physiological level, referring to neurons 'cathected with a certain quantity [of energy]' [2], systems '*loaded *with a sum of excitation' [17] and '*provided *with a quota of affect' [18]. Succinctly, the term 'cathexis' means 'libidinal investment'. It is a vitally important concept for the integration of Freudian metapsychology with principles of modern neuroscience. In this paper, we discuss changes in haemodynamic response and other neurophysiological measures in relation to the withdrawal and investment of libido.

### Object cathexis

The concept of "the object" is used in a broad sense in psychoanalysis to refer to literal, abstract and symbolic objects. People, tasks, work and ideas can all serve as objects. The process of object cathexis can be compared with the process of goal-directed cognition, since both require libidinal investment. Based on neuroimaging data in depression (see Neuropsychiatric findings in depression correlated with principles of Freudian metapsychology below), we propose that activation of the dorsolateral prefrontal cortex (DLPFC) correlates with object cathexis, and reduced DLPFC activation correlates with reduced object cathexis which manifests in depression as anhedonia (see Hypofrontality below). As will be discussed in the next section, activation of the DLPFC is accompanied by a deactivation in a network of regions known as the default-mode network (DMN) [19]. The DMN is highly active during resting cognition. The regions engaged during active cognition are referred to here as the object-oriented network (ON). We propose that activation in the ON and deactivation in the DMN correlates with the process of object cathexis.

### The ego

The German original 'das Ich' literally translates as 'the I'. It is somewhat regrettable that Freud's terms have not been translated more literally since the originals have an appeal that is lost in translation. Freud used the concept of the ego in a number of different ways; a useful way of gaining a sense of the different applications therefore, is to cite some examples of its use:

1. A referent to the conscious sense of self:

' [I]n each individual there is a coherent organisation of mental processes; and this we call his ego. It is to this ego that consciousness is attached' [1].

2. An unconscious force maintaining self-cohesion:

'It is certain that much of the ego is itself unconscious and notably what we may call its nucleus; only a small part of it is covered by the term "preconscious"' [20].

3. A nucleus of somatic cohesion:

'The ego is first and foremost a bodily ego' [1].

4. A reservoir of libido:

'Thus we form the idea of there being an original libidinal cathexis of the ego, from which some is later given off to objects' [7].

'The ego is the true and original reservoir of libido' [20].

5. The primary agent of repression:

' [T]he ego is the power that sets repression in motion' [12].

**Figure 1:**
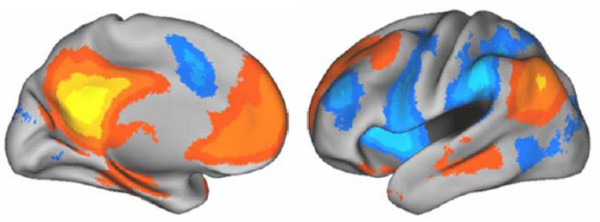
**Regions positively correlated with the default mode network (orange), most notably the medial prefrontal cortex (mPFC), posterior cingulate cortex (PCC), inferior parietal lobule and medial temporal regions**. Activity in these regions has been shown to decrease during the performance of goal-directed cognition. The areas shown in blue are negatively correlated with the default mode network (DMN) and may be described as an object-oriented network (ON). The ON is consistently activated during goal-directed cognitions but is relatively inactive at rest. It is argued in the present work that the DMN is functionally consistent with the Freudian ego. Image reproduced with permission from [289].

Given the many different functions to the ego, it would be counterintuitive to suggest that it is 'housed' in a single given region of the brain. Based on a large number of neuroimaging studies, we propose that a highly connected network of regions, principally incorporating the medial prefrontal cortex (mPFC), posterior cingulate cortex (PCC), inferior parietal lobule (IPL) and medial temporal regions [19,21-31] meets many of the criteria of the Freudian ego. This conglomeration of activity has been named the 'default mode network' [19] (Figure 1). A recent analysis in a large sample of healthy volunteers has shown that connectivity within the DMN undergoes a marked increase with maturation from childhood to adulthood [31]. Activity in the mPFC node of the DMN has been closely associated with self-reflection (e.g. [22,24,27,32]) and recent evidence suggests that the mPFC exerts the principal causality within the network [33]. The PCC and IPL have been associated with proprioception [34,35] and the PCC and medial temporal regions have been associated with the retrieval of autobiographical memories [36-39]. The DMN shows a high level of functional connectivity at rest [28,33]. Activity in this network consistently decreases during engagement in goal-directed cognition [28,33,40] and connectivity within the network tends to decrease during states of reduced consciousness [41,42]. Expressed in Freudian terms, goal-directed cognition requires a displacement of libido (energy) from the ego's reservoir (the DMN) and its investment in objects (activation of the DLPFC). There is evidence that this function is impaired in a number of psychiatric disorders, including depression [43-48].

'The ego is a great reservoir from which the libido that is destined for objects flows out and into which it flows back from those objects' [49].

In addition to the mPFC and PCC nodes of the DMN and their relation to the ego, we speculate on the basis of neuroimaging data and findings from deep brain stimulation (see Neuropsychiatric findings in depression correlated with principles of Freudian metapsychology below), that ventromedial PFC (vmPFC) exerts a strong repressive hold over emotional and motivational ('visceromotor') centres [50]. This repressive force is the most primitive function of the ego. As will be elaborated later, the posterior vmPFC plays a major role in the pathophysiology of depression. For example, inhibition of the region ventral to the genu of the copus callosum, the subgenual cingulate or Cg25 has been found to alleviate depressive symptomology in patients suffering from treatment resistant depression (TRD) [51]. The subgenual cingulate and regions proximal to it appear to exert a modulatory influence over key 'visceromotor' centres such as the amygdala, the ventral tegmental area (VTA) and the nucleus accumbens (NAc) [50,52]. Certain limbic centres (e.g., the amygdala) have been shown to be pathologically active in depression (see [50] for a review).

### The ego ideal/super ego

The concept of the 'ego ideal' was introduced by Freud in his paper 'On narcissism' [7], forming the basis of what would later become 'the super ego' [1] (German original = 'Das über-Ich'; 'the over-I'). The ego ideal/super ego plays a fundamental role in the aetiology of depression:

'Repression, we have said, proceeds from the ego, we might say with greater precision that it proceeds from the self-respect of the ego' [7].

Freud described this more fully in the following passage:

'The ego ideal is...the target of the self-love which was enjoyed in childhood by the actual ego. The subject's narcissism makes its appearance displaced on to this new ideal ego, which like the infantile ego finds itself possessed of every perfection that is of value. As always where the libido is concerned, man has here shown himself incapable of giving up a satisfaction he had once enjoyed. He is not willing to forgo the narcissistic perfection of his childhood; and when as he grows up, he is disturbed by the admonitions of others and by the awakening of his own critical judgement, so that he can no longer retain that perfection, he seeks to recover it in the new form of an ideal. What he projects before him as his ideal is the substitute for the lost narcissism of his childhood in which he was his own ideal' [7].

It is difficult to postulate a neurodynamic correlate of such a high-level concept as the ego ideal or super ego. The following model should therefore be considered speculative and preliminary. The super ego might be thought of as an umbrella term for high-level cognitions that work to appraise the ego's ability to meet an imagined ideal. This ideal-ego or 'ego ideal' is acquired through an internalisation of value judgements of others (e.g., one's early care givers) under social and environmental demands (see Mourning and melancholia below). Through the super ego, the ego receives feedback on how closely it corresponds with an imagined ideal. If the super ego judges the ego as falling short of this ideal, or if the super ego judges the ego's or the id's drives as unhealthy or dangerous in the context of its social environment, then the ego may repel these drives, withholding them from consciousness. The implications of the super ego's instruction to repress will be discussed in the next section in relation to depression.

It is highly unlikely that the ego ideal/super ego is housed in any specific region of the brain but we may speculate about dynamic physiological processes paralleling psychological ones. Thus, paralleling the super ego's value judgements of the ego may be feedback between the DLPFC of the ON and the mPFC of the DMN. Information communicated between these two systems (see The ego above) may parallel the experience of pursuing an ideal and judging how successfully it is met.

In relation to the unconscious, punishing aspect of the super-ego it might be useful to consider the role of the anterior cingulate (ACC). Activation of the ACC has been associated with error detection and guilt [8,53,54]. It may be significant that a recent analysis of functional connectivity in the human cingulate revealed strong connectivity between the ACC and the DLPFC [54]. Conversely, Cg25 was found to be strongly connected with regions of the DMN such as the OFC. It is possible that feedback between the DLPFC and the mPFC is mirrored at a lower level by feedback between the ACC, OFC and Cg25. Feedback between the ON and DMN likely takes place via cortico-striato-pallido-thalamo-cortical circuitry.

The super ego's control over the ego gives it a unique power to influence the motility and expression of the drives. Impassioned behaviours deemed dangerous to the ego in the context of its environment may be denied expression by activating Cg25 and the DMN. Integrating this hypothesis into a model of depression, we can postulate that activating Cg25 and the DMN controls the full expression of affective, mnemonic and motivational behaviours promulgated by visceromotor centres. Thus, engaging Cg25 contains limbic activity within paralimbic-thalamic circuits maintained by the Cg25 in reaction to sustained limbic arousal (for relevant models, see [46,50,55-58]).

### The id

The German original 'das es' literally translates as 'the it'. As with the German word for the ego (das Ich), the original word for the id has an appeal that is lost in translation. The id was one of Freud's later concepts, being introduced in his paper 'The ego and the id' [1]. Some have argued that the id is synonymous with the unconscious, and it is true that two are closely related:

'The id and the unconscious are as intimately linked as the ego and the preconscious' [59].

'The truth is that it is not only the psychically repressed that remains alien to our consciousness, but also some of the impulses which dominate our ego' [6].

Although the id and the unconscious are related, they also retain some important differences, both psychologically and physiologically. Essentially, the id refers to the unconscious as a system in a topographical sense [60]. Freud described the id as an archaic psychical system governed by primitive drives.

'We now distinguish in our mental life (which we regard as an apparatus compounded of several agencies, districts or provinces) one region which we call the *ego *proper and another which we name the *id*. The id is the older of the two; the ego has developed out of it, like a cortical layer, through the influence of the external world. It is in the id that all our primary drives are at work, all the processes in the id take place unconsciously' [61].

The function of the id corresponds closely with that of the mesocorticolimbic dopamine system [62]. The NAc and VTA are especially sensitive to rewarding stimuli [63]. Neuroimaging studies in humans have shown that rewarding stimuli activate dopaminergic cells in the VTA [64-66] eliciting an increase of dopamine release in the NAc [67]. Jaak Panksepp has described the mesocorticolimbic dopamine system as the appetitive, motivational or 'seeking' system [68]. High voltage electrical stimulation of the NAc in both animals and humans has been found to elicit pleasurable and sexual responses [68,69] and ejaculation in human males has been found to correlate with activation of the VTA [64].

### The unconscious

James Strachey explained in a footnote to Freud's paper 'The unconscious' [6] that the German word for 'unconscious' ('*das unbewusste*') typically translates as 'not consciously known' and does not have the unhelpful connotation of the English equivalent meaning 'knocked out' or 'comatose'. This information is useful for an understanding of this difficult concept. Along with repression, the theory of a conscious/unconscious dynamic is one of the most important in psychoanalysis. The term unconscious is used in both a topographical ('the system unconscious') and descriptive sense (e.g., 'rendered unconscious') [60]. When we speak of 'the unconscious', it is usually the topographical meaning that is being employed. In this paper, we refer to 'the unconscious' as an archaic psychical system with its own characteristic phenomenology and physiology.

James Uleman comments in the introduction to the book 'The new unconscious' [70] that 'the psychoanalytic unconscious is widely acknowledged to be a failure as a scientific theory because evidence of its major components cannot be observed, measured precisely, or manipulated easily'. In order to address this not unreasonable charge, it is important for those who have 'turned their ear' to the unconscious to devise a method of demonstrating its phenomenology to those who have not. A case will be made in this paper that the study of consistent phenomenologies in a number of different altered states of consciousness such as dreaming, acute psychotic states, the aura of temporal lobe epilepsy and psychedelic drug induced states will provide converging evidences for the existence of a characteristic psychical system. It is hoped that identifying the neurophysiological activity paralleling the subjective phenomena in these states will provide the necessary scientific breakthrough to finally do away with the persuasive impression that the unconscious does not exist.

Identifying the correlates of 'primary process' (see The primary and secondary psychical process below) activities taking place during wakefulness is extremely difficult given the relatively rigid, impervious nature of normal waking consciousness. The altered states of consciousness mentioned above are comparatively much more yielding. For example, during transient episodes of 'dreamlike' cognition, the normal processes of repression may be disturbed, allowing unconscious material to flow into consciousness with greater freedom. In a recent review of human intracranial electroencephalography recordings of rapid eye movement (REM) sleep, acute psychotic states, temporal lobe auras and psychedelic drug states, Carhart-Harris identified bursts of rhythmic theta and slow-wave activity in the medial temporal regions in all these states and hypothesised that these discharges of limbic theta are the signature activity of the unconscious mind, described by Freud as 'the primary psychical process' [71].

### The primary and secondary psychical process

'We have found that processes in the unconscious or in the id obey different laws from those in the preconscious ego. We name these laws in their totality the *primary process*, in contrast to the secondary process which governs the course of events in the preconscious, in the ego' [59].

Dating back to his early work on dissociative states [72], Freud described two distinct laws or principles governing the distribution of psychical energy in the mind: (1) *the secondary psychical process *of normal waking consciousness which exerts a tonic inhibitory hold over the primary psychical process in accordance with the demands of social context; (2) The archaic and ontogenetically and phylogenetically regressive *primary psychical process*. The primary psychical process describes the relatively motile, free-flowing activity of the unconscious mind. The primary psychical process becomes observable when the forces of repression are circumvented by the forces of the unconscious. Such episodes are characterised by a fluidity of association – perceptually and cognitively, and a flooding of affect.

This paper takes the position that discharges of rhythmic theta and slow-wave activity from the medial temporal lobes to the association cortices are the signature activity of the primary psychical process of the unconscious mind [71].

### Repression

Freud described repression in the following ways:

'The theory of repression is the corner-stone on which the whole structure of psycho-analysis rests' [7].

' [T]he essence of repression lies simply in turning something away, and keeping it at a distance, from the conscious' [6].

' [R]epression is brought to bear invariably on ideas which evoke a distressing affect (unpleasure) in the ego' [2].

'The repressions behave like dams against the pressure of water' [73].

'The mechanisms of repression...[involve] a *withdrawal of the cathexis of energy *(or of *libido*)' [6].

**Figure 2:**
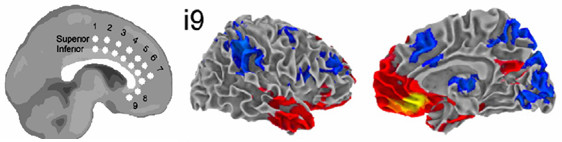
**Functional connectivity of the subgenual cingulate (Cg25)**. Yellow/red indicates regions positively correlated with the seed region (i9) and blue indicates regions negatively correlated with the seed region. The seed region, i9, fell within the area of Cg25. This region's network of connectivity incorporated several areas associated with the default mode network (DMN). Although it is not clear in these images, activity in Cg25 was also strongly correlated with activity in the ventral striatum and medial temporal regions. Image reproduced with permission [54].

Based on the evidence reviewed below, we propose that the Cg25, the orbitofrontal cortex (OFC) and vmPFC exert a strong repressive hold over visceromotor centres, serving to restrain untempered drive and flurries of unconscious material from discharging into the cortices and being consciously registered (Figure 2). It is likely however that there are different gradations of repression and that the repressive function takes place more through a set of processes than the action of a specific nucleus. We maintain that Cg25 exerts the principal suppressive effect on visceromotor centres but it is likely that the vmPFC and OFC facilitate this action (see The function of the vmPFC and OFC in relation to repression below). We also speculate that the repressive function is modulated by information transmitted through feedback between the ON and the DMN (see The ego idea/super ego above).

'For the ego, the formation of an ideal would be the conditioning factor for repression' [7].

## Mourning and melancholia

In 'Mourning and melancholia' [74], Freud compared the experience of mourning with the pathological state of depression:

'It is well worth notice that, although mourning involves grave departures from the normal attitude to life, it never occurs to us to regard it as a pathological condition and refer to it medical treatment. We rely on it being overcome after a certain lapse of time, and we look upon any interference with it as useless or even harmful. The distinguishing mental features of melancholia, are a profoundly painful sense of dejection, a cessation of interest in the outside world, loss of capacity to love, inhibition of all activity...a lowering of the self-regarding feelings to a degree that finds utterance in self-reproaches and self-revilings, and culminates in a delusional expectation of punishment' [74].

Freud described how both mourning and depression involve a forced withdrawal of object cathexis. Since this withdrawal is involuntary, it is experienced as a painful process against which the ego protests. The ego denies the loss and strives to place within its grasp a substitute object – whether real or imaginary, in fantasy or hallucination. In cases of successful recovery, the energetic ties which once bound the subject to the object begin to be severed and the libidinal energies that flowed out of the ego and into the object are displaced into alternative objects.

In depression, the attempted recovery begins in a similar manner to mourning, with a protest from the ego and search for a substitute object. However, failing to find a suitable replacement in the outside world and refusing to concede that the object is lost, the ego draws within itself its own cathexes. The energies, which were before sent out freely from the ego, now return from the object to condense and concentrate upon it.

'Thus the shadow of the object fell upon the ego' [74].

In depression, this is experienced as an increase in introspection and a reciprocal decrease in interest in the outside world. The ego, having taken itself as its own object, begins a process of self-evaluation. The self-questioning becomes fiercely critical as ambivalent feelings felt towards the lost object and self-rapprochement for failing to live up to ideals are targeted at the ego.

'The object cathexis...was brought to an end. But the free libido was not displaced onto another object; it was withdrawn into the ego. There, however, it was not employed in an unspecified way, but served to establish an *identification *of the ego with the abandoned object. Thus, the shadow of the object fell upon the ego, and the latter could henceforth be judged by a special agency, as though it were an object, the forsaken object. In this way an object-loss was transformed into an ego-loss and the conflict between the ego and the loved person into a cleavage between the critical activity of the ego and the ego as altered by identification' [74].

Object loss in mourning relates to a literal death; the psychological significance of which is well appreciated by the mourner and those around him/her. Accordingly, expressions of sadness in mourning are viewed as appropriate, healthy and cathartic. In depression however, the negative affect that accompanies the condition is often viewed as disproportionate to the individual's circumstances – both by the individual him/herself and by others. In contrast to mourning, Freud argued that the intense, ostensibly disproportionate level of negative affect experienced in depression is symptomatic of unpleasant and problematic emotions (e.g., love and resentment) that are denied a fully conscious actualisation:

' [In depression], one cannot see clearly what it is that has been lost, and it is all the more reasonable to suppose that the patient cannot consciously perceive what he has lost either. This, indeed, might be so even if the patient is aware of the loss that has given rise to his melancholia, but only in the sense he knows *whom *he has lost but not *what *he has lost in him. This would suggest that melancholia is in some way related to an object-loss which is withdrawn from consciousness, in contradistinction to mourning, in which there is nothing about the loss that is unconscious' [74].

If we are to be consistent with Freud's economic theory of libido [2], the intensity of the mental anguish experienced in depression is proportionate to the intensity of the emotion held back from consciousness, and the severity of aggression directed towards the self is proportionate to the severity of aggression that, were it not for repression, would be propelled towards the object:

'Ambivalence gives a pathological cast to mourning and forces it to express itself in the form of self-reproaches to the effect that the mourner himself is to blame for the loss of the loved object, i.e., that he has willed it... If the love for the object – a love which cannot be given up though the object itself is given up – takes refuge in narcissistic identification, then the hate comes into operation on this substitutive object, abusing it, debasing it, making it suffer and deriving sadistic satisfaction from its suffering... It is sadism alone that solves the riddle of the tendency to suicide, which makes the melancholic so interesting – and so dangerous. So immense is the ego's self-love, which we have come to recognise as the primal state from which instinctual life proceeds, and so vast is the amount of narcissistic libido that we see liberated in the threat to life, that we cannot conceive how the ego can consent to its own destruction. We have known, it is true, that no neurotic harbours thoughts of suicide which he has not turned back upon himself from murderous impulses against others' [74].

In addition to the anger and resentment that is turned towards the ego, the ego is admonished for failing to live up to expectations. 'Mourning and melancholia' was written shortly after Freud introduced the idea of 'the ego ideal' [17] that would later become 'the super ego' [1]. As discussed in section 1.5, the super ego is a critical agency that judges the ego in relation to its own ideal.

'The melancholic displays something else besides which is lacking in mourning – an extraordinary diminution in his self-regard, an impoverishment of his ego on a grand scale. In mourning it is the world that has become poor and empty; in melancholia it is the ego itself. The patient represents his ego to us as worthless, incapable of any achievement and morally despicable; he reproaches himself, vilifies himself and expects to be punished. He abases himself before everyone and commiserates with his own relatives for being connected with someone so unworthy' [74].

The super ego is of central importance in psychoanalytic theory, but it is a much more difficult concept to identify physiologically than e.g., libido or cathexis. Freud argued that the super ego results from a process that took place in infancy (the Oedipus complex) as a recapitulation of a process that occurred in the development of the species [75]. Through this process, the infant was coerced via parental and communal authority to renounce its libidinal demands. Although the infant's free reign was put to an end, he/she internalised the demands for concession and turned them into an image of an ideal:

'The broad general outcome of the sexual phase dominated by the Oedipus complex may, therefore, be taken to be the forming of a precipitate in the ego, consisting of these two identifications in some way united with each other. This modification of the ego retains its special position; it confronts the other contents of the ego as an ego ideal or super ego' [1].

'The super ego retains the character of the father, the more powerful the Oedipus complex was and the more rapidly it succumbed to repression (under the influence of authority, religious teaching, schooling and reading), the stricter will be the domination of the super ego over the ego later on – in the form of conscience or perhaps of an unconscious sense of guilt' [1].

' [I]n the undertaking of repression, the ego is at bottom following the commands of its super ego – commands which, in their turn, originate from influences in the external world that have found representation in the super ego. The fact remains that the ego *has *taken sides with those powers, that in it their demands have more strength than the instinctual demands of the id, and that the ego is the power that sets the repression in motion against the portion of the id concerned' [1].

To summarise the key processes involved in depression as outlined by Freud: the illness is triggered by the loss of an object imbued with a particularly intense level of libidinal cathexis, there is a forced withdrawal of cathexis, a regression of libido into the ego, a critical judgement of the ego based on its failure to live up to ideals, and a simultaneous attacking of the ego by repressed emotions felt towards the lost object.

' [Melancholias] show us the ego divided, fallen apart into two pieces, one which rages against the second. This second piece is the one which has been altered by introjection and which contains the lost object. But the piece that behaves so cruelly is not unknown to us either. It comprises the conscience, a critical agency within the ego, which even in normal times takes up a critical attitude towards the ego, though never so relentlessly and so unjustifiably' [76].

## Neuropsychiatric findings in depression correlated with principles of Freudian metapsychology

### Hypofrontality

**Figure 3:**
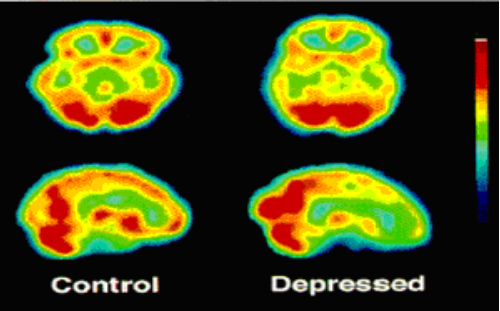
**Single photon emission computed tomography (SPECT) images from a depressed patient showing characteristic hypofrontality relative to a healthy control subject**[82].

One of the most consistent findings in the neuroimaging of depression is decreased cerebral blood flow (CBF) and glucose metabolism in the PFC, particularly the DLPFC [77-85] (figure 3). The PFC is a large and functionally heterogeneous structure. Studies of frontal activity in depression have highlighted these differences, with the DLPFC, associated with cognitive and executive functions showing decreased activity in depressed states, and the ventral PFC, associated with emotional processing, showing increased activity during episodes of emotional rumination (see [86] or [50]).

Several studies have found negative correlations between depression severity and frontal metabolism [78,81,87-93]. The induction of depressed symptomology in healthy volunteers and remitted depressed patients has been found to correlate reliably with decreases in frontal activity [56,94,95]. Frontal blood flow and metabolism tends to normalise after spontaneous or treatment-induced remission [51,78,79,96-105]. These studies highlight the reliability of frontal hypometabolism, particularly in the DLPFC, in neuroimaging studies of depression.

Based on the neuroimaging data we speculate that hypoactivity in the DLPFC is a correlate of withdrawn object cathexis experienced subjectively as impoverished motivation and diminished interest in the matters outside of the self. A recent functional magnetic resonance imaging (fMRI) study reported a positive correlation between subjective measures of anhedonia and activity in the vmPFC and OFC (Brodmann areas (BA)10, 11, and 32) [106]. Importantly, an additional relationship was found between anhedonia scores and diminished activation of the amygdala and the ventral striatum. As will be explained in the following section, in depression, Cg25 can be envisaged as functioning in a manner analogous to a dam, preventing ascending energies from being invested in the PFC.

' [T]he ego controls the approaches to motility – that is, to the discharge of excitations into the external world...' [107].

### Hyperactivity and electrical stimulation of Cg25

**Figure 4:**
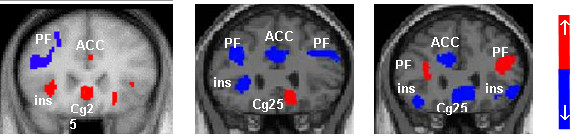
**Positron emission tomography (PET) images of cerebral blood flow changes during transient induced sadness in healthy controls (left); pre deep brain stimulation (DBS) in depressed patients (centre); and 3-month post DBS in treatment responsive patients (right)**. Hyperactivity in Cg25 and hypoactivity in the dorsolateral prefrontal cortex (DLPFC) is evident during low mood and depression. This situation is reversed during remission of symptoms. ACC, anterior cingulate cortex; ins = insular; PF, prefrontal cortex [51,95].

Certainly one of the most exciting findings in neuropsychiatry in recent years has been the identification of Cg25 as a key region in the pathophysiology of depression. Several neuroimaging studies have correlated hyperactivity in this region with depressed mood states and induced sadness in healthy volunteers and depressed patients [46,56,95,107-114] (figure 4). Depression severity is correlated with Cg25 hypermetabolism [115] and increased functional connectivity [46]. Spontaneous and treatment-induced remission of symptoms is associated with significantly decreased Cg25 metabolism [51,100,105,110,113,116-119].

The subgenual cingulate has been the target of ablative surgeries in the past [120] and, more recently, DBS [51], where high frequency stimulation is used to inhibit activity in target nuclei. The preliminary results of chronic bilateral high frequency stimulation of Cg25 in six patients suffering from severe treatment-resistant depression were reported by Mayberg and colleagues [51]. Significant improvements (a 50% or greater reduction in Hamilton depression rating scale (HDRS-17) score) were seen in five of the six patients at 2-month follow-up with sustained improvements achieved in four patients at 6 months. Positron emission tomography (PET) scans of patients at 3 and 6 months post stimulation revealed decreased blood flow in Cg25 and increased blood flow in the DLPFC. Significant improvements were seen in sleep, energy, interest and psychomotor speed. Patients and their families reported 'renewed interest and pleasure in social and family activities, decreased apathy and anhedonia, as well as improved ability to plan, initiate, and complete tasks that were reported as impossible prior to surgery'.

At the 2007 international *Neuropsychoanalysis *congress in Vienna, some first-person accounts relating to acute stimulation were reported:

'It isn't like something has been added – no, something has been taken away'.

'It is as if I have just suddenly shifted from a state of all consuming internal focus to realising that there are number of things around to do'.

'When you're depressed the focus is inwards. So if someone tells you, well you aren't the only one who feels like that, you don't care. With the stimulator, I don't feel that inward look; it has lifted so I am not so focused on myself...'.

'It is as though I have been locked in a room with 10 screaming children; constant noise, no rest, no escape. Whatever just happened, the children have just left the building'

The 'something...taken away' described in these accounts is consistent with the idea of a release from repression (deactivation of Cg25) and a return to object cathexis (DLPFC activation). The final account is especially interesting given that the patient was a father of 5.

Interestingly, sudden and dramatic deactivations of Cg25 and functionally related regions of the vmPFC and OFC have recently been recorded after intravenous infusion of the dissociative hallucinogen ketamine in healthy human volunteers [121]. These deactivations correlated strongly with dissociative phenomena. Significant activations were seen in the parahippocampal gyrus, temporal cortex and PCC. Importantly, the regions deactivated by ketamine (OFC and vmPFC) are those postulated in this paper to be involved in the process of repression, and the regions activated by ketamine (specifically the medial temporal structures), are those we hypothesise to be involved in the primary psychical process of the unconscious mind. As with the classic psychedelic drugs (e.g., LSD and psilocybin), the effects of ketamine have been described as disturbing the mechanisms of repression and facilitating the release of primary process thought [122]. Single doses of ketamine have been found to elicit a short-term antidepressant effect in depressed patients [123-126] and the drug has also been used as an adjunct to psychotherapy with reported efficacy in the treatment of alcoholism [122].

### The function of the vmPFC and OFC in relation to repression

The data cited in the previous section supports the hypothesis that Cg25 plays a key role in repression. However, it is likely that Cg25 does not act alone in this regard. For example, activity in the ventral anterior PFC correlates positively with depression severity and activity in this region decreases after effective treatment [50]. The OFC (BA11 and BA47) is activated when subjects try to decrease arousal to erotic films [127] and there is impoverished activation of BA10 and 11 in paedophile sex offenders viewing paedophilic material [128]. In healthy controls viewing the same images, the lateral OFC (BA47) was activated. The lateral OFC has also been found to be activated during contemplation of moral transgressions [129] and script-induced guilt [130].

Using autobiographical scripts designed to evoke strong emotion, healthy control subjects showed increased blood flow in the vmPFC during script-induced anger compared to patients with anger attacks who showed an impoverished vmPFC response [131]. Impoverished vmPFC activation in anger patients suggests that recruitment of this region is necessary for suppression of aggressive affect. Significantly lower resting metabolism has been recorded in the OFC of patients with a history of reactive aggression [132,133] and patients with OFC and mPFC lesions who show an increased risk of reactive aggression [134-136]. Healthy participants who imagined responding in an unrestrained aggressive manner to an assault showed hypoactivity in the OFC but increased activity in the same region when imagining restraint [137]. In cases of post traumatic stress disorder, a condition characterised by unsuccessful repression of traumatic memories, patients exposed to a script-driven reminder of a personally traumatic experience showed impoverished activity in the rostral anterior cingulate compared with controls [138]. A related study showed a strong negative correlation between emotional scores and vmPFC activation in PTSD patients exposed to script-driven reminders of their traumatic experience [139] implying that impoverished vmPFC activation facilitates the return of the affect attached to the original trauma.

As will be discussed in the next section, activation of the amygdala is associated with the expression of primitive emotions such as anger and fear as well as complex autobiographical recollections [140,141]. It is interesting therefore that the study by Dougherty and colleagues cited above discovered an inverse relationship between blood flow in the vmPFC and amygdala in healthy control subjects during script-driven anger but a positive relationship between amygdala and vmPFC activity in patients with anger attacks [131]. These findings imply that patients with anger attacks suffer from ineffective suppression of amygdala activation [131]. A number of studies have demonstrated that activation of the amygdala with concomitant emotional arousal is very quickly followed by activation of the OFC [142-145] and – in healthy individuals – suppression of the amygdala response [146-148]. This suppressive function of the vmPFC/OFC is supported by a large body of preclinical data [147-160]. It is likely that this function is impaired in depression, with the suppressive/repressive action of the vmPFC/OFC being dominated by persistent flurries of limbic arousal [161].

### Amygdala hyperactivity and electrical stimulation of medial temporal lobes

Hyperactivity in the amygdala has been reported in a large number of imaging studies of depression [47,87,97,111,162-169]. Increased activity in the amygdala has been recorded in studies of induced sadness in healthy volunteers [169-171]. Amygdala activity has been found to correlate positively with depression severity [87,162,166], to show a sustained response to negative emotional stimuli in depressed patients compared to healthy controls [161] and to decrease in sensitivity to emotional stimuli after successful antidepressant treatment [172,173].

The amygdala has long been recognised to play an important role in emotion. Bilateral resection of the amygdala has been found to result in dramatic behavioural changes (Klüver and Bucy syndrome) including emotional blunting, indifference to loved ones, hyperorality and hypersexuality [140]. Electrical stimulations of the human amygdala and medial temporal regions have been found to elicit a range of primitive emotional responses including: fear, anxiety, anger, aggression, sexual behaviours, déjà vu and autobiographical recollections [141,174-185]:

'I just get the electrical feeling, and it goes all the way through me...it makes me do things I don't want to do – I get mad' [10].

'I had a flash of familiar memory, but I don't know what it was... I had a little memory – a scene in a play. They were talking and I could see it... Just seeing it in my memory...a very familiar memory of a girl talking to me...that feeling of familiarity – a familiar memory' [177].

A thorough phenomenological review of these experiences is necessary for an appreciation of the functional significance of the medial temporal lobes in relation to the primary psychical process of the unconscious mind. Such experiences have been interpreted by several clinicians and researchers as examples of primary process activity taking over from the secondary psychical process of normal waking consciousness [72,174,177,179,180,186-193]:

'Reflected in the seizure-related behaviour may be emotional trauma of early life, negative feelings towards specific individuals because of past incidents or situations' [192].

'Repression fails, the usual defence systems crumble, disturbing unconscious material erupts, anxiety mounts, and the personality structure becomes ineffective' [189].

'It is in my view wrong to call the feeling of having experienced something before an illusion. It is rather that at such moments something is touched on which we have already experienced once before' [5].

A review of depth electroencephalography recordings from the medial temporal regions suggests that stimulation-induced dreamlike experiences share a common phenomenology and neurophysiology (bursts of rhythmic theta and slow-wave activity) with other dreamlike states [71]. It is hoped that converging evidences correlating neurophysiological activity with qualitatively analysed phenomenological experiences will facilitate a wider understanding of the phenomenology and psychophysiology of the unconscious mind.

### Cg25 connectivity

Anatomical studies in primates have revealed dense connections between Cg25 and the hypothalamus [194,195] mPFC [196], parahippocampal cortex [197], amygdala, ventral striatum, septal nuclei, dorsomedial caudate nucleus and mediodorsal nucleus of the thalamus; with moderate connections to the periaqueductal grey and dorsal raphe nucleus [195,196]. Human tractography and functional connectivity analyses support these findings, showing prominent connections between Cg25 and the NAc, amygdala, hypothalamus, OFC and vmPFC [54,198,199]. The connections of Cg25 to a number of important visceromotor centres offering profuse projections to the PFC has led to suggestions that Cg25 plays an important modulatory role in cortical functioning [195]. A recent cytological analysis of the human cingulate cortex has revealed an especially dense concentration of inhibitory receptors in Cg25 [200]. These findings are consistent with the hypothesis that Cg25 exerts a controlling influence over visceromotor regions.

Connectivity between Cg25 and the amygdala has been found to be especially strong during the viewing of fearful and threatening faces [201]. The magnitude of disconnectivity between these structures predicted anxiety scores in a number of individuals [201]. Resting state connectivity between Cg25 and a range of structures including the medial temporal lobes has been found to predict treatment response in depressed patients [58] and a strong correlation was discovered between subjective measures of neuroticism and Cg25 and amygdala activation during the viewing of emotionally provocative images [202].

In addition to medial temporal structures, other important visceromotor centres connected with Cg25 include the NAc [198,203,204] and the VTA [196,205]. Cg25 shows an especially high level of functional connectivity with the NAc at rest [23,54,204]. The NAc and VTA are key nuclei in the mesocorticolimbic dopamine systems, being central to the mechanisms of motivation and reward [10,68]. The VTA has also been found to be strongly activated during ejaculation in male humans [64] cocaine induced euphoria [65] and the heroin rush [66]. Electrical stimulation of the NAc and the septum has been found to elicit feelings of sexual pleasure and orgasm in humans [69,206,207]. Patients given a self-stimulator connected to the septal region stimulated themselves repeatedly for hours and protested bitterly when attempts were made to take the device from them [206]. These findings support the association of the mesolimbic dopamine system with the Freudian 'id' [1]. Chronic stimulation of the NAc has recently been carried out in three TRD patients [208]. Early results suggest that this intervention may be particularly effective in relieving symptoms of anhedonia.

### Volumetric reductions

'The neurosis may last a considerable time and cause marked disturbances, but it may also run a latent course and be overlooked. As a rule defence retains the upper hand in it; in any case alterations of the ego, comparable to scars, are left behind' [61].

Postmortem and MRI studies have found glial loss and volume reductions in the PFC in major depressive disorder (MDD) and bipolar disorder (BPD) [209-214] as well as extensive losses in Cg25 and proximal paralimbic regions [163,201,215-221]. Unilateral and bilateral volumetric reductions in the medial temporal regions – primarily in the hippocampus, have also been reported in depressed patients [112,201,222-232], as have reductions in the ventral striatum [233,234].

It is not difficult to surmise that the metabolic work of repression has structural ramifications. This paper hypothesises that the volumetric reductions found in postmortem and neuroimaging studies of depression are related to the effects of repression. It is significant that the most severe reductions have been found in Cg25 (48% reductions in 163) the area hypothesised to exert the primary repressive force. One possible mechanism for the volumetric reductions is glucocorticiod-mediated neurotoxicity [235]. Dysregulation of the stress related hypothalamic-pituitary-adrenal (HPA) axis is consistently associated with depression [236]. Dysregulation of the HPA may be related to hyperactivity in the amygdala [237]. Electrical stimulation of the amygdala increases cortisol release in humans [238]. HPA hyperactivity increases the likelihood of excitotoxic processes, downregulating glial, and increasing the concentrations of neurotoxic glucocorticiods and excitiotoxic glutamate [239]. The OFC and the vmPFC are dense in glucocorticiod receptors and glutamate cells, with glutamatergic afferents ascending from the amygdala and hippocampus [240].

It is hypothesised that the volumetric reductions discovered in depressed patients, as well as patients suffering from other major psychiatric conditions such as schizophrenia [194,208,210,212,215,216,219,241-248] may be related to the effects of psychological conflict.

## Discussion

The main inspiration behind the primary hypothesis of this paper i.e., that Cg25 is centrally involved in repression, was Mayberg's paper on DBS for the treatment of severe depression [51]. The findings of this study have a special significance for Freudian metapsychology. It has been inferred in this paper that the sudden lifting of negative affect upon stimulation of Cg25 is consistent with the idea of a release of libido for object cathexis after it has been pathologically 'dammed up' behind a central repressing force. However, the therapeutic response to stimulation raises some difficult questions for both psychoanalysis and psychiatry. One important question concerns the economy of psychical energy in relation to depression and mania. Freud discussed the issue of mania towards the end of 'Mourning and melancholia':

'The impression which several psycho-analytic investigators have already put into words is that the content of mania is no different from that of melancholia, that both disorders are wrestling with the same "complex", but that probably in melancholia the ego has succumbed to the complex whereas in mania it has mastered it or pushed it aside. Our second pointer is afforded by the observation that all states such as joy, exultation or triumph, which give us the normal model for mania, depend on the same economic conditions. What has happened here is that, as a result of some influence, a large expenditure of psychical energy, long maintained or habitually occurring, has at last become unnecessary, so that it is available for numerous applications and possibilities of discharge' [74].

Neuroimaging studies of manic patients have shown that in direct contrast to depression, resting state activity is decreased in the OFC [249,250] and increased in dorsal frontal areas during manic episodes [250,251]. It is significant that the early responses to Cg25 stimulation do not appear to switch patients from pathological depression to overt mania. According to Freud's model, manic episodes depend on a quantity of dammed-up libido being suddenly made available for object cathexis.

'An important element in the theory of repression is the view that repression is not an event that occurs once but that it requires a permanent expenditure [of energy]. If this expenditure were to cease, the repressed impulse, which is being fed all the time from its sources, would on the next occasion flow along the channels from which it had been forced away, and the repression would either fail in its purpose or would have to be repeated an indefinite number of times. Thus it is because drives are continuous in their nature that the ego has to make its defensive action secure by a permanent expenditure [of energy]' [252].

In the long term it is feasible that abeyance of repression leads to a therapeutic shift in the energetic equilibrium of the mind; but even if this is true, we still need to consider why upon release from repression, we do not see a pathological release of primitive drive and repressed memory. Are we to assume that electrical stimulation of Cg25 removes both the physiological *and *psychological causes of depression? Even if depression is primarily an energetic phenomenon and the physiological causes *are *the psychological causes (and *vice versa*), wouldn't there still remain memory traces and exogenous stressors facilitating a recall of lost objects, regretted behaviours and eluded ideals?

One possible reason why the effect of Cg25 stimulation appears to have a sustained beneficial effect [253] rather than an iatrogenic one [254] may be that inhibiting activity in Cg25 facilitates the disintegration of a wider network. For example, it is possible that activation of Cg25 supports activation of the DMN and deactivation of Cg25 supports deactivation of the DMN and activation of the ON. This model would account for the diminished self focus (ego cathexis) and rejuvenated task focus (object cathexis) seen upon Cg25 stimulation. It may be possible to test this formulation through neuroimaging studies of patients undergoing Cg25 stimulation. If the ego is dependent on repression, we would expect to see decreased activity in the DMN, decreased activity in Cg25 and increased activity in the ON after stimulation. Preliminary evidence lends support to this model [51]. Evidence supporting the interdependency of the ego and repression would of course have important implications for the history of the evolution of human consciousness.

In order to test the validity of the Freudian model, it is important that there be thorough psychophenomenological and neurophysiological analyses of Cg25 and NAc stimulations. Ideally, subjective and objective measures should be taken simultaneously, in real time. If, as this paper predicts, Cg25 is centrally involved in repression, then in addition to dramatic improvements in energy/libido we would also expect to see some adverse responses to stimulation, such as disinhibited behaviour, pathological drive, perseverance, hostility, aggression, sexual promiscuity and a reduced capacity to consider others. Such behaviours are commonly associated with ventromedial prefrontal lesions [255-259]. We would also predict that patients undergoing Cg25 stimulation would be more susceptible to temporal lobe phenomena (such as déjà vu) as a result of diminished inhibitory control over excitatory medial temporal structures. If electrophysiological recordings are carried out, we would hypothesise that electrodes placed within the proximity of septal, supramammillary or hippocampal theta structures would display characteristic bursts of high voltage rhythmic theta during moments of strong emotion ([69], see [71] for a review). We would also predict that intracranial EEG recordings in bipolar patients would reveal significant changes in activity paralleling shifts in mood i.e., Cg25 hyperactivity/NAc hypoactivity during depression and Cg25 hypoactivity/NAc hyperactivity during mania.

It is acknowledged that very little in the way of counter evidence has been cited in this paper challenging the validity of the Freudian model. It is likely that several examples could be found in Freud's evolving work of hypotheses that do not correspond well with the findings of modern clinical research. However, it must be emphasised that what we have brought together in this paper are principal concepts of Freudian metapsychology together with principal findings of neuropsychiatry. It is all the more significant therefore that the meeting has been complementary.

In order to develop a discussion of the comparative merits of psychological paradigms, it is worth reminding ourselves of the two main aims of this paper: (1) to propose a series of hypotheses correlating neurophysiological processes with some fundamental processes of psychoanalysis, and (2) to highlight correspondences between Freud's writings in 'Mourning and melancholia' and current findings in depression. How successful these tasks have been will largely depend on two factors: (1) whether evidences from other fields converge with the evidences reviewed here, and (2) whether the psychoanalytic perspective is given credence. There is already ample evidence to support the role of Cg25, the vmPFC and OFC in suppressing primitive affect, but the psychoanalytic significance of this function has yet to be fully appreciated.

It has been said before that it matters little which psychological discipline we choose to derive our operational terms; the approach is secondary to the phenomena:

'Listen my friend, the golden tree of life is green, all theory is grey' [260].

Psychological models do serve a purpose however, but to provide comprehensive explanations of mental states and behaviours, effective models must evolve naturally from their phenomena. In a recent letter published in a reputable journal and co-signed by a number of leading researchers [261] a proposal was put-forward as part of a 'decade of the mind' initiative to work towards a transdisciplinary explanation of mental phenomena. The main psychological discipline championed by the authors was cognitive psychology. While the essential idea is a commendable one, we must ask ourselves seriously whether the information processing paradigm is really the best model for carrying out this initiative. The psychological limitations of the behavioural model have been recognised for several decades but the cognitive approach, which views the human mind as an information processor is currently the favoured model of clinicians and researchers. If the computer analogy is an accurate representation of the human psyche, then we can feel comfortable going into the final years of the 'decade of the mind' that real progress will be made. If however, the model is at all incomplete, we may need to consult alternative paradigms to assist our empiricism. The information processing model has traditionally been put to good use guiding and informing empirical research. However, several researchers are now recognising that the computational model has limitations, especially when it is applied to human emotion [62,68,262,263]. What we hope psychoanalysis can bring to the table therefore, is a psychological model that has its roots set firmly in human experience. We hope psychoanalysis can work alongside cognitive psychology to provide a more comprehensive understanding of human experience.

' [T]he mind would often slip through the fingers of psychology, if psychology refused to keep a hold on the mind's unconscious states' [264].

'Psychoanalysis still represents the most coherent and intellectually satisfying view of the mind that we have' [263].

The primary requirement for a scientific psychoanalysis is (and always has been) to confirm beyond reasonable doubt that the unconscious mind exists and that it is not only important but *essential *for an understanding of the human mind and behaviour. If, as this paper maintains, the unconscious does exist, then regardless of the words chosen to define it, the establishment of its phenomenology as subject matter worthy of scientific investigation is important. Deciding how best to test and confirm the hypothesis that the unconscious mind exists will present us simultaneously with a direction towards studying its form and physiology. Due to the rigour of repression, depression is not the easiest phenomenon to gain a perspective on the workings of the unconscious. The psychoses provide a better vantage:

'Things that in the neuroses have to be laboriously fetched up from the depths are found in the psychoses on the surface, visible to every eye' [265].

' [M]aterial which is ordinarily unconscious can transform itself into preconscious material and then become conscious – a thing that happens to a large extent in psychotic states. From this we infer that the maintenance of certain internal resistances is a *sine qua non *of normality' [59].

In depression we only assume the existence of the unconscious through a process of deduction based on ostensibly irrational behaviours (e.g., self-harm, violent self-criticism etc). As Freud made clear, there are much better ways of studying the unconscious and the free-flowing psychical energies that are its signature. Freud first stumbled across a realisation of the unconscious through his work on dissociative states [72]:

' [O]ne received the clearest impression – especially from the behaviour of subjects *after *hypnosis – of the existence of mental processes that one could only describe as "unconscious". The "unconscious" has it is true, long been under discussion among philosophers as a theoretical concept; but now for the first time, in the phenomena of hypnotism, it became something actual, tangible and subject to experiment' [1].

'To most people educated in philosophy the idea of anything psychical which is not also conscious is so inconceivable that it seems to them absurd and refutable simply by logic. I believe this is only because they have never studied the relevant phenomena of hypnosis and dreams, which – quite apart from pathological manifestations – necessitate this view. Their psychology of consciousness is incapable of solving the problems of dreams and hypnosis' [1].

For Freud, dreams were a way of studying the unconscious – unfettered by waking consciousness but the phenomenology of dreaming has largely failed to convince sceptics of the existence of the unconscious. Freud acknowledged that converging lines of enquiry would be required to consolidate the insights gained through the study of dreams:

'Thus, the psychological hypotheses to which we are led by an analysis of the process of dreaming must be left, as it were, in suspense, until they can be related to the findings of other enquiries which seek to approach the kernel of the same problem from another angle' [5].

Future work may provide the necessary evidence. Alternative means of studying the unconscious – perhaps by way of a pharmacological agent such as a psychedelic drug [193,266] may open up fresh angles of enquiry. Freud famously described the interpretation of dreams as 'the royal road to a knowledge of the unconscious activities of the mind' [5]. However, dreaming occurs in sleep, making real-time recitation of subjective phenomena impossible. If we could stimulate the primary psychical process in waking we would have a more effective method for studying the unconscious:

'Freud once said of dreams that they were the *via regia *or royal way to study the unconscious; to an even greater degree this seems to be true for the LSD experience' [265].

It is anticipated that progress towards a wider appreciation of the psychoanalytic model will first require confirmation of the existence of the unconscious mind. We propose that the most effective way of achieving this is to stimulate the primary psychical process in waking consciousness. There is a wealth of evidence to suggest that this can be reliably achieved through the use of a psychedelic drug such as LSD [193,266-285]:

'One must...put it simply, it does seem that all LSD does is open the doors to the unconscious' [279].

Using neuroimaging techniques we would predict that the ego dissolving, primary process releasing properties of a psychedelic compound would correspond with a shift in effective connectivity in the DMN, with the medial temporal regions (as opposed to the vmPFC) exerting principal causality [33,121,286,287]. Testing this hypothesis will be difficult, but such challenging procedures are necessary if the primary psychical process is to be considered a matter worthy of investigation. Once we are better able to study the phenomenology of the unconscious, the application of our new knowledge to the study and treatment of the whole of the mind will follow more easily.

## Conclusion

The goal of this paper has been to investigate consistencies between Freudian metapsychology and empirical findings in neuropsychiatry. A summary of several key psychoanalytic concepts was given together with some early hypotheses about their physiological coordinates. This was done to facilitate an understanding of Freudian terminology and allow for the application of these ideas to areas of clinical interest. Modern clinical research and older empirical work such as intracranial stimulations was discussed in relation to Freudian metapsychology in order to highlight correspondences between physiology, phenomenology and theory. If a new level of scientific verification is achieved for subjective phenomena of relevance to psychoanalysis, this will have implications not just for the way in which psychoanalysis is viewed by the wider philosophical, psychological and psychiatric communities, but also for those interested in incorporating psychoanalytic ideas into their own clinical practice.

' [I]t should not be forgotten that in fact [the distinction between the unconscious and conscious dimensions of the mind] is not a theory at all but a first stock-taking of the facts of our observations, that it keeps as close to them as possible' [59].

'*Psycho-analysis an Empirical Science *– Psychoanalysis is not, like philosophies, a system starting out from a few sharply defined basic concepts, seeking to grasp the whole universe with the help of these and, once it is completed, having no room for fresh discoveries or better understanding. On the contrary, it keeps close to the facts in its field of study, seeks to solve the immediate problems of observation, gropes its way forward by the help of experience, is always incomplete and always ready to correct or modify its theories. There is no incongruity (anymore than in the case of physics or chemistry) if its most general concepts lack clarity and if its postulates are provisional; it leaves their more precise definition to the results of future work' [288].

## Competing interests

The authors declare that they have no competing interests.

## Authors' contributions

The present paper was inspired by the work of HSM as presented at the 2007 International Neuropsychoanalysis congress in Vienna. The paper was written by RLC–H with intellectual support and guidance from HSM, ALM and DJN. All authors read and approved the final manuscript.

## References

[B1] Freud S (1923). The ego and the id.

[B2] Freud S (1895). Project for a scientific psychology.

[B3] Pribram K, Gill M (1976). Freud's project re-assessed.

[B4] Strachey J, Strachey J (1954). Freud S (1886–1899) Project for a scientific psychology.

[B5] Freud S (1900). The interpretation of dreams.

[B6] Freud S (1915). The unconscious.

[B7] Freud S (1914). On narcissism.

[B8] Fletcher P, McKenna PJ, Friston KJ, Frith CD, Dolan RJ (1999). Abnormal cingulate modulation of fronto-temporal connectivity in schizophrenia. Neuroimage.

[B9] Freud S (1891). On aphasia.

[B10] MacLean PD (1990). The triune brain in evolution.

[B11] Fonagy P (2003). Psychoanalysis today. World Psychiatry.

[B12] Freud S (1924). Neurosis and psychosis.

[B13] Freud S (1894). Draft F.

[B14] Freud S (1905). Three essays on the theory of sexuality.

[B15] Jung CG (1928). On psychic energy. On the nature of the psyche.

[B16] Strachey J, Strachey J (1924). Freud S (1893–1899) The emergence of Freud's fundamental hypotheses.

[B17] Freud S (1894). The psychoneuroses of defence.

[B18] Freud S (1893). Some points for an organic study of hysterical and motor paralyses.

[B19] Raichle ME, MacLeod AM, Snyder AZ, Powers WJ, Gusnard DA, Shulman GL (2001). A default mode of brain function. Proc Natl Acad Sci USA.

[B20] Frued (1920). Beyond the pleasure principle.

[B21] Gusnard DA, Raichle ME (2001). Searching for a baseline: functional imaging and the human brain. Nat Rev Neurosci.

[B22] Gusnard DA, Akbudak E, Shulman GL, Raichle ME (2001). Medial prefrontal cortex and self-referential mental activity: relation to a default mode of brain function. Proc Natl Acad Sci USA.

[B23] Greicius MD, Krasnow B, Reiss AL, Menon V (2003). Functional connectivity in the resting brain: a network analysis of the default mode hypothesis. Proc Natl Acad Sci USA.

[B24] Johnson SC, Baxter LC, Wilder LS, Pipe JG, Heiserman JE, Prigatano GP (2002). Neural correlates of self-reflection. Brain.

[B25] Vogeley K, May M, Ritzl A, Falkai P, Zilles K, Fink GR (2004). Neural correlates of first-person perspective as one constituent of human self-consciousness. J Cogn Neurosci.

[B26] Kelley WM, Macrae CN, Wyland CL, Caglar S, Inati S, Heatherton TF (2002). Finding the self? An event-related fMRI study. J Cogn Neurosci.

[B27] Fossati P, Hevenor SJ, Graham SJ, Grady C, Keightley ML, Craik F, Mayberg H (2003). In search of the emotional self: an fMRI study using positive and negative emotional words. Am J Psychiatry.

[B28] Fox MD, Snyder AZ, Vincent JL, Corbetta M, Van Essen DC, Raichle ME (2005). The human brain is intrinsically organized into dynamic, anticorrelated functional networks. Proc Natl Acad Sci USA.

[B29] Fransson P (2005). Spontaneous low-frequency BOLD signal fluctuations: an fMRI investigation of the resting-state default mode of brain function hypothesis. Hum Brain Map.

[B30] Greicius MD, Supekar K, Menon V, Dougherty RF (2008). Resting-state functional connectivity reflects structural connectivity in the default mode network. Cereb Cortex.

[B31] Fair DA, Cohen AL, Dosenbach NU, Church JA, Miezin FM, Barch DM, Raichle ME, Petersen SE, Schlaggar BL (2008). The maturing architecture of the brain's default network. Proc Natl Acad Sci USA.

[B32] Buckner RL, Carroll DC (2006). Self-projection and the brain. Trends Cogn Sci.

[B33] Uddin LQ, Clare Kelly AM, Biswal BB, Xavier Castellanos F, Milham MP (2008). Functional connectivity of default mode network components: correlation, anticorrelation, and causality. Hum Brain Map.

[B34] Saxe R, Xiao DK, Kovacs G, Perrett DI, Kanwisher N (2004). A region of right posterior superior temporal sulcus responds to observed intentional actions. Neuropsychologia.

[B35] Stark M, Coslett H, Saffran E (1996). Impairment of an egocentric map of locations: Implications for perception and action. Cogn Neuropsychol.

[B36] Maddock RJ, Garrett AS, Buonocore MH (2001). Remembering familiar people: the posterior cingulate cortex and autobiographical memory retrieval. Neurosci.

[B37] Maguire EA, Mummery CJ (1999). Differential modulation of a common memory retrieval network revealed by positron emission tomography. Hippocampus.

[B38] Vincent JL, Snyder AZ, Fox MD, Shannon BJ, Andrews JR, Raichle ME, Buckner RL (2006). Coherent spontaneous activity identifies a hippocampal-parietal memory network. J Neurophysiol.

[B39] Gilboa A, Winocur G, Grady CL, Hevenor SJ, Moscovitch M (2004). Remembering our past: functional neuroanatomy of recollection of recent and very remote personal events. Cereb Cortex.

[B40] Raichle ME, Snyder AZ (2007). A default mode of brain function: a brief history of an evolving idea. Neuroimage.

[B41] Greicius MD, Kiviniemi V, Tervonen O, Vainionpää V, Alahuhta S, Reiss AL, Menon V (2008). Persistent default-mode network connectivity during light sedation. Hum Brain Map.

[B42] Rombouts SA, Barkhof F, Goekoop R, Stam CJ, Scheltens P (2005). Altered resting state networks in mild cognitive impairment and mild Alzheimer's disease: an fMRI study. Hum Brain Map.

[B43] Kennedy DP, Redcay E, Courchesne E (2006). Failing to deactivate: resting functional abnormalities in autism. Proc Natl Acad Sci USA.

[B44] Garrity AG, Pearlson GD, McKiernan K, Lloyd D, Kiehl KA, Calhoun VD (2007). Aberrant "default mode" functional connectivity in schizophrenia. Am J Psychiatry.

[B45] Zhou Y, Liang M, Tian L, Wang K, Hao Y, Liu H, Liu Z, Jiang T (2007). Functional disintegration in paranoid schizophrenia using resting-state fMRI. Schizophr Res.

[B46] Greicius MD, Flores BH, Menon V, Glover GH, Solvason HB, Kenna H, Reiss AL, Schatzberg AF (2007). Resting-state functional connectivity in major depression: abnormally increased contributions from subgenual cingulate cortex and thalamus. Biol Psychiatry.

[B47] Anand A, Li Y, Wang Y, Wu J, Gao S, Bukhari L, Mathews VP, Kalnin A, Lowe MJ (2005). Activity and connectivity of brain mood regulating circuit in depression: a functional magnetic resonance study. Biol Psychiatry.

[B48] Pomarol-Clotet E, Salvador R, Sarró S, Gomar J, Vila F, Martínez A, Guerrero A, Ortiz-Gil J, Sans-Sansa B, Capdevila A, Cebamanos JM, McKenna PJ (2008). Failure to deactivate in the prefrontal cortex in schizophrenia: dysfunction of the default mode network?. Psychol Med.

[B49] Freud S (1917). A difficulty in the path of psycho-analysis.

[B50] Drevets WC (2007). Orbitofrontal cortex function and structure in depression. Ann NY Acad Sci.

[B51] Mayberg HS, Lozano AM, Voon V, McNeely HE, Seminowicz D, Hamani C, Schwalb JM, Kennedy SH (2005). Deep brain stimulation for treatment-resistant depression. Neuron.

[B52] Dougherty DD, Shin LM, Rauch SL, Zald DH, Rauch SL (2006). Orbitofrontal cortex activation during functional neuroimaging studies of emotion induction in humans. The orbitofrontal cortex.

[B53] Meyer-Lindenberg AS, Olsen RK, Kohn PD, Brown T, Egan MF, Weinberger DR, Berman KF (2005). Regionally specific disturbance of dorsolateral prefrontal-hippocampal functional connectivity in schizophrenia. Arch Gen Psychiatry.

[B54] Margulies DS, Kelly AM, Uddin LQ, Biswal BB, Castellanos FX, Milham MP (2007). Mapping the functional connectivity of anterior cingulate cortex. Neuroimage.

[B55] Mayberg HS, Brannan SK, Mahurin RK, Jerabek PA, Brickman JS, Tekell JL, Silva JA, McGinnis S, Glass TG, Martin CC, Fox PT (1997). Cingulate function in depression: a potential predictor of treatment response. Neuroreport.

[B56] Mayberg HS, Liotti M, Brannan SK, McGinnis S, Mahurin RK, Jerabek PA, Silva JA, Tekell JL, Martin CC, Lancaster JL, Fox PT (1999). Reciprocal limbic-cortical function and negative mood: converging PET findings in depression and normal sadness. Am J Psychiatry.

[B57] Mayberg HS (2003). Modulating dysfunctional limbic-cortical circuits in depression: towards development of brain-based algorithms for diagnosis and optimised treatment. Br Med Bull.

[B58] Seminowicz DA, Mayberg HS, McIntosh AR, Goldapple K, Kennedy S, Segal Z, Rafi-Tari S (2004). Limbic-frontal circuitry in major depression: a path modeling metanalysis. Neuroimage.

[B59] Freud S (1940). An outline of psychoanalysis.

[B60] Freud S (1933). New introductory lectures of psychoanalysis.

[B61] Freud S (1939). Moses and monotheism.

[B62] Solms M, Turnbull O (2002). The brain and the inner world.

[B63] Pagnoni G, Zink CF, Montague PR, Berns GS (2002). Activity in human ventral striatum locked to errors of reward prediction. Nat Neurosci.

[B64] Holstege G, Georgiadis JR, Paans AM, Meiners LC, Graaf FH van der, Reinders AA (2003). Brain activation during human male ejaculation. J Neurosci.

[B65] Breiter HC, Gollub RL, Weisskoff RM, Kennedy DN, Makris N, Berke JD, Goodman JM, Kantor HL, Gastfriend DR, Riorden JP, Mathew RT, Rosen BR, Hyman SE (1997). Acute effects of cocaine on human brain activity and emotion. Neuron.

[B66] Sell LA, Morris J, Bearn J, Frackowiak RS, Friston KJ, Dolan RJ (1999). Activation of reward circuitry in human opiate addicts. Eur J Neurosci.

[B67] Pappata S, Dehaene S, Poline JB, Gregoire MC, Jobert A, Delforge J, Frouin V, Bottlaender M, Dolle F, Di Giamberardino L, Syrota A (2002). *In vivo *detection of striatal dopamine release during reward: a PET study with [(11)C]raclopride and a single dynamic scan approach. Neuroimage.

[B68] Panksepp J (1998). Affective neuroscience.

[B69] Heath RG (1964). The role of pleasure in behavior.

[B70] Hassin RR, Uleman JS, Bargh JA (2005). The new unconscious.

[B71] Carhart-Harris R (2007). Waves of the unconscious: The neurophysiology of dreamlike states and its implications for the psychodynamic model of the mind. Neuropsychoanalysis.

[B72] Breuer J, Freud S Studies on hysteria Standard edition of the complete works of Sigmund Freud.

[B73] Freud S (1937). Analysis terminable and interminable.

[B74] Freud S (1917). Mourning and melancholia.

[B75] Freud S (1913). Totem and taboo.

[B76] Freud S (1921). Group psychology and the analysis of the ego.

[B77] Bremner JD, Innis RB, Salomon RM, Staib LH, Ng CK, Miller HL, Bronen RA, Krystal JH, Duncan J, Rich D, Price LH, Malison R, Dey H, Soufer R, Charney DS (1997). Positron emission tomography measurement of cerebral metabolic correlates of tryptophan depletion-induced depressive relapse. Arch Gen Psychiatry.

[B78] Baxter LR, Schwartz JM, Phelps ME, Mazziotta JC, Guze BH, Selin CE, Gerner RH, Sumida RM (1989). Reduction of prefrontal cortex glucose metabolism common to three types of depression. Arch Gen Psychiatry.

[B79] Bench CJ, Friston KJ, Brown RG, Scott LC, Frackowiak RS, Dolan RJ (1992). The anatomy of melancholia – focal abnormalities of cerebral blood flow in major depression. Psychol Med.

[B80] Biver F, Goldman S, Delvenne V, Luxen A, De Maertelaer V, Hubain P, Mendlewicz J, Lotstra F (1994). Frontal and parietal metabolic disturbances in unipolar depression. Biol Psychiatry.

[B81] Cohen RM, Gross M, Nordahl TE, Semple WE, Oren DA, Rosenthal N (1992). Preliminary data on the metabolic brain pattern of patients with winter seasonal affective disorder. Arch Gen Psychiatry.

[B82] Mayberg HS, Lewis PJ, Regenold W, Wagner HN (1994). Paralimbic hypoperfusion in unipolar depression. J Nucl Med.

[B83] Ebert D, Feistel H, Barocka A (1991). Effects of sleep deprivation on the limbic system and the frontal lobes in affective disorders: a study with Tc-99m-HMPAO SPECT. Psychiatry Res.

[B84] Ring HA, Bench CJ, Trimble MR, Brooks DJ, Frackowiak RS, Dolan RJ (1994). Depression in Parkinson's disease. A positron emissionstudy. Br J Psychiatry.

[B85] Drevets WC, Ongür D, Price JL (1998). Reduced glucose metabolism in the subgenual prefrontal cortex in unipolar depression. Mol Psychiatry.

[B86] Dougherty DD, Rauch SL (2007). Brain correlates of antidepressant treatment outcome from neuroimaging studies in depression. Psychiatr Clin North Am.

[B87] Drevets WC, Videen TO, Price JL, Preskorn SH, Carmichael ST, Raichle ME (1992). A functional anatomical study of unipolar depression. J Neurosci.

[B88] Yazici KM, Kapucu O, Erbas B, Varoglu E, Gülec C, Bekdik CF (1992). Assessment of changes in regional cerebral blood flow in patients with major depression using the 99mTc-HMPAO single photon emission tomography method. Eur J Nucl Med.

[B89] Andreason PJ, Altemus M, Zametkin AJ, King AC, Lucinio J, Cohen RM (1992). Regional cerebral glucose metabolism in bulimia nervosa. Am J Psychiatry.

[B90] Hirono N, Mori E, Ishii K, Ikejiri Y, Imamura T, Shimomura T, Hashimoto M, Yamashita H, Sasaki M (1998). Frontal lobe hypometabolism and depression in Alzheimer's disease. Neurology.

[B91] Mayberg HS, Starkstein SE, Sadzot B, Preziosi T, Andrezejewski PL, Dannals RF, Wagner HN, Robinson RG (1990). Selective hypometabolism in the inferior frontal lobe in depressed patients with Parkinson's disease. Ann Neurol.

[B92] Volkow ND, Hitzemann R, Wang GJ, Fowler JS, Wolf AP, Dewey SL, Handlesman L (1992). Long-term frontal brain metabolic changes in cocaine abusers. Synapse.

[B93] Bonne O, Krausz Y, Shapira B, Bocher M, Karger H, Gorfine M, Chisin R, Lerer B (1996). Increased cerebral blood flow in depressed patients responding to electroconvulsive therapy. J Nucl Med.

[B94] Bremner JD, Vythilingam M, Ng CK, Vermetten E, Nazeer A, Oren DA, Berman RM, Charney DS (2003). Regional brain metabolic correlates of alpha-methylparatyrosine-induced depressive symptoms: implications for the neural circuitry of depression. JAMA.

[B95] Liotti M, Mayberg HS, McGinnis S, Brannan SL, Jerabek P (2002). Unmasking disease-specific cerebral blood flow abnormalities: mood challenge in patients with remitted unipolar depression. Am J Psychiatry.

[B96] Drevets WC (1998). Functional neuroimaging studies of depression: the anatomy of melancholia. Ann Rev Med.

[B97] Drevets WC, Raichle ME (1992). Neuroanatomical circuits in depression: implications for treatment mechanisms. Psychopharmacol Bull.

[B98] Goodwin GM, Austin MP, Dougall N, Ross M, Murray C, O'Carroll RE, Moffoot A, Prentice N, Ebmeier KP (1993). State changes in brain activity shown by the uptake of 99mTc-exametazime with single photon emission tomography in major depression before and after treatment. J Affect Disord.

[B99] Martinot JL, Hardy P, Feline A, Huret JD, Mazoyer B, Attar-Levy D, Pappata S, Syrota A (1990). Left prefrontal glucose hypometabolism in the depressed state: a confirmation. Am J Psychiatry.

[B100] Nobler MS, Sackeim HA, Prohovnik I, Moeller JR, Mukherjee S, Schnur DB, Prudic J, Devanand DP (1994). Regional cerebral blood flow in mood disorders, III. Treatment and clinical response. Arch Gen Psychiatry.

[B101] Rubin P, Hemmingsen R, Holm S, Møller-Madsen S, Hertel C, Povlsen UJ, Karle A (1994). Relationship between brain structure and function in disorders of the schizophrenic spectrum: single positron emission computerized tomography, computerized tomography and psychopathology of first episodes. Acta Psychiatr Scand.

[B102] Trivedi MH, Morris DW, Grannemann BD, Mahadi S (1994). Symptom clusters as predictors of late response to antidepressant treatment. J Clin Psychiatry.

[B103] Mayberg HS (1997). Limbic-cortical dysregulation: a proposed model of depression. J Neuropsychiatry Clin Neurosci.

[B104] Ogura A, Morinobu S, Kawakatsu S, Totsuka S, Komatani A (1998). Changes in regional brain activity in major depression after successful treatment with antidepressant drugs. Acta Psychiatr Scand.

[B105] Kennedy SH, Evans KR, Krüger S, Mayberg HS, Meyer JH, McCann S, Arifuzzman AI, Houle S, Vaccarino FJ (2001). Changes in regional brain glucose metabolism measured with positron emission tomography after paroxetine treatment of major depression. Am J Psychiatry.

[B106] Keedwell PA, Andrew C, Williams SC, Brammer MJ, Phillips ML (2005). The neural correlates of anhedonia in major depressive disorder. Biol Psychiatry.

[B107] Baker SC, Frith CD, Dolan RJ (1997). The interaction between mood and cognitive function studied with PET. Psychol Med.

[B108] George MS, Ketter TA, Parekh PI, Horwitz B, Herscovitch P, Post RM (1995). Brain activity during transient sadness and happiness in healthy women. Am J Psychiatry.

[B109] Bench CJ, Friston KJ, Brown RG, Frackowiak RS, Dolan RJ (1993). Regional cerebral blood flow in depression measured by positron emission tomography: the relationship with clinical dimensions. Psychol Med.

[B110] Pardo JV, Sheikh SA, Schwindt GC, Lee JT, Kuskowski MA, Surerus C, Lewis SM, Abuzzahab FS, Adson DE, Rittberg BR (2008). Chronic vagus nerve stimulation for treatment-resistant depression decreases resting ventromedial prefrontal glucose metabolism. Neuroimage.

[B111] Drevets WC, Price JL, Bardgett ME, Reich T, Todd RD, Raichle ME (2002). Glucose metabolism in the amygdala in depression: relationship to diagnostic subtype and plasma cortisol levels. Pharmacol Biochem Behav.

[B112] Videbech P, Ravnkilde B (2004). Hippocampal volume and depression: a meta-analysis of MRI studies. Am J Psychiatry.

[B113] Dougherty DD, Weiss AP, Cosgrove GR, Alpert NM, Cassem EH, Nierenberg AA, Price BH, Mayberg HS, Fischman AJ, Rauch SL (2003). Cerebral metabolic correlates as potential predictors of response to anterior cingulotomy for treatment of major depression. J Neurosurg.

[B114] Nofzinger EA, Buysse DJ, Germain A, Price JC, Meltzer CC, Miewald JM, Kupfer DJ (2005). Alterations in regional cerebral glucose metabolism across waking and non-rapid eye movement sleep in depression. Arch Gen Psychiatry.

[B115] Osuch EA, Ketter TA, Kimbrell TA, George MS, Benson BE, Willis MW, Herscovitch P, Post RM (2000). Regional cerebral metabolism associated with anxiety symptoms in affective disorder patients. Biol Psychiatry.

[B116] Liotti M, Martin CC, Gao JH, Roby JW, Mayberg HS, Zamarripa F, Jerabek PA, Fox PT (1997). Xenon effects on regional cerebral blood flow assessed by 15O-H2O positron emission tomography: implications for hyperpolarized xenon MRI. J Mag Res Imag.

[B117] Wu J, Buchsbaum MS, Gillin JC, Tang C, Cadwell S, Wiegand M, Najafi A, Klein E, Hazen K, Bunney WE, Fallon JH, Keator D (1999). Prediction of antidepressant effects of sleep deprivation by metabolic rates in the ventral anterior cingulate and medial prefrontal cortex. Am J Psychiatry.

[B118] Mayberg HS, Brannan SK, Tekell JL, Silva JA, Mahurin RK, McGinnis S, Jerabek PA (2000). Regional metabolic effects of fluoxetine in major depression: serial changes and relationship to clinical response. Biol Psychiatry.

[B119] Mayberg HS, Silva JA, Brannan SK, Tekell JL, Mahurin RK, McGinnis S, Jerabek PA (2002). The functional neuroanatomy of the placebo effect. Am J Psychiatry.

[B120] Cosgrove GR, Rauch SL (1995). Psychosurgery. Neurosurg Clin N Am.

[B121] Deakin JF, Lees J, McKie S, Hallak JE, Williams SR, Dursun SM (2008). Glutamate and the neural basis of the subjective effects of ketamine: a pharmaco-magnetic resonance imaging study. Arch Gen Psychiatry.

[B122] Krupitsky EM, Grinenko AY (1997). Ketamine psychedelic therapy (KPT): a review of the results of ten years of research. J Psychoactive Drugs.

[B123] Berman RM, Cappiello A, Anand A, Oren DA, Heninger GR, Charney DS, Krystal JH (2000). Antidepressant effects of ketamine in depressed patients. Biol Psychiatry.

[B124] Ostroff R, Gonzales M, Sanacora G (2005). Antidepressant effect of ketamine during ECT. Am J Psychiatry.

[B125] Zarate CA, Singh JB, Carlson PJ, Brutsche NE, Ameli R, Luckenbaugh DA, Charney DS, Manji HK (2006). A randomized trial of an N-methyl-D-aspartate antagonist in treatment-resistant major depression. Arch Gen Psychiatry.

[B126] Liebrenz M, Borgeat A, Leisinger R, Stohler R (2007). Intravenous ketamine therapy in a patient with a treatment-resistant major depression. Swiss Med Week.

[B127] Beauregard M, Lévesque J, Bourgouin P (2001). Neural correlates of conscious self-regulation of emotion. J Neurosci.

[B128] Schiffer B, Paul T, Gizewski E, Forsting M, Leygraf N, Schedlowski M, Kruger TH (2008). Functional brain correlates of heterosexual paedophilia. Neuroimage.

[B129] Finger EC, Marsh AA, Kamel N, Mitchell DG, Blair JR (2006). Caught in the act: the impact of audience on the neural response to morally and socially inappropriate behavior. Neuroimage.

[B130] Shin LM, Dougherty DD, Orr SP, Pitman RK, Lasko M, Macklin ML, Alpert NM, Fischman AJ, Rauch SL (2000). Activation of anterior paralimbic structures during guilt-related script-driven imagery. Biol Psychiatry.

[B131] Dougherty DD, Rauch SL, Deckersbach T, Marci C, Loh R, Shin LM, Alpert NM, Fischman AJ, Fava M (2004). Ventromedial prefrontal cortex and amygdala dysfunction during an anger induction positron emission tomography study in patients with major depressive disorder with anger attacks. Arch Gen Psychiatry.

[B132] Raine A, Meloy JR, Bihrle S, Stoddard J, LaCasse L, Buchsbaum MS (1998). Reduced prefrontal and increased subcortical brain functioning assessed using positron emission tomography in predatory and affective murderers. Behav Sci Law.

[B133] Goyer PF, Andreason PJ, Semple WE, Clayton AH, King AC, Compton-Toth BA, Schulz SC, Cohen RM (1994). Positron-emission tomography and personality disorders. Neuropsychopharmacology.

[B134] Grafman J, Schwab K, Warden D, Pridgen A, Brown HR, Salazar AM (1996). Frontal lobe injuries, violence, and aggression: a report of the Vietnam Head Injury Study. Neurology.

[B135] Anderson SW, Bechara A, Damasio H, Tranel D, Damasio AR (1999). Impairment of social and moral behavior related to early damage in human prefrontal cortex. Nat Neurosci.

[B136] Eslinger PJ, Grattan LM (1994). Altered serial position learning after frontal lobe lesion. Neuropsychologia.

[B137] Pietrini P, Guazzelli M, Basso G, Jaffe K, Graffman J (2000). Neural correlates of imaginal aggressive behaviour assessed by positron emission tomography in healthy subjects. Am J Psychiatry.

[B138] Britton JC, Phan KL, Taylor SF, Fig LM, Liberzon I (2005). Corticolimbic blood flow in posttraumatic stress disorder during script-driven imagery. Biol Psychiatry.

[B139] Frewen P, Lane RD, Neufeld RW, Densmore M, Stevens T, Lanius R (2007). Neural correlates of levels of emotional awareness during trauma script-imagery in posttraumatic stress disorder. Psychosom Med.

[B140] Terzian H, Ore GD (1955). Syndrome of Kluver and Bucy. Reproduced in man by bilateral removal of the temporal lobes. Neurology.

[B141] Gloor P (1990). Experiential phenomena of temporal lobe epilepsy. Brain.

[B142] Kawasaki H, Kaufman O, Damasio H, Damasio AR, Granner M, Bakken H, Hori T, Howard MA, Adolphs R (2001). Single-neuron responses to emotional visual stimuli recorded in human ventral prefrontal cortex. Nat Neurosci.

[B143] Krolak-Salmon P, Hénaff MA, Vighetto A, Bertrand O, Mauguière F (2004). Early amygdala reaction to fear spreading in occipital, temporal, and frontal cortex: a depth electrode ERP study in humans. Neuron.

[B144] Streit M, Ioannides AA, Liu L, Wölwer W, Dammers J, Gross J, Gaebel W, Müller-Gärtner HW (1999). Neurophysiological correlates of the recognition of facial expressions of emotion as revealed by magnetoencephalography. Brain Res Cogn Brain Res.

[B145] Streit M, Ioannides A, Sinnemann T, Wölwer W, Dammers J, Zilles K, Gaebel W (2001). Disturbed facial affect recognition in patients with schizophrenia associated with hypoactivity in distributed brain regions: a magnetoencephalographic study. Am J Psychiatry.

[B146] Garcia R, Vouimba RM, Baudry M, Thompson RF (1999). The amygdala modulates prefrontal cortex activity relative to conditioned fear. Nature.

[B147] Hariri AR, Bookheimer SY, Mazziotta JC (2000). Modulating emotional responses: effects of a neocortical network on the limbic system. Neuroreport.

[B148] Milad MR, Quirk GJ (2002). Neurons in medial prefrontal cortex signal memory for fear extinction. Nature.

[B149] Morgan MA, Romanski LM, LeDoux JE (1993). Extinction of emotional learning: contribution of medial prefrontal cortex. Neurosci Lett.

[B150] Morgan MA, LeDoux JE (1995). Differential contribution of dorsal and ventral medial prefrontal cortex to the acquisition and extinction of conditioned fear in rats. Behav Neurosci.

[B151] LeDoux JE (1996). The emotional brain.

[B152] LeDoux JE (2000). Emotion circuits in the brain. Ann Rev Neurosci.

[B153] Quirk GJ, Russo GK, Barron JL, Lebron K (2000). The role of ventromedial prefrontal cortex in the recovery of extinguished fear. J Neurosci.

[B154] LeDoux JE, Gorman JM (2001). A call to action: overcoming anxiety through active coping. Am J Psychiatry.

[B155] Garcia R (2002). Postextinction of conditioned fear: between two CS-related memories. Learn Mem.

[B156] Grace AA, Rosenkranz JA (2002). Regulation of conditioned responses of basolateral amygdala neurons. Physiol Behav.

[B157] Herry C, Garcia R (2002). Prefrontal cortex long-term potentiation, but not long-term depression, is associated with the maintenance of extinction of learned fear in mice. J Neurosci.

[B158] Harrison BJ, Pujol J, Ortiz H, Fornito A, Pantelis C, Yücel M (2008). Modulation of brain resting-state networks by sad mood induction. PLoS ONE.

[B159] Myers KM, Davis M (2002). Behavioral and neural analysis of extinction. Neuron.

[B160] Paré D, Quirk GJ, Ledoux JE (2004). New vistas on amygdala networks in conditioned fear. J Neurophysiol.

[B161] Siegle GJ, Steinhauer SR, Thase ME, Stenger VA, Carter CS (2002). Can't shake that feeling: event-related fMRI assessment of sustained amygdala activity in response to emotional information in depressed individuals. Biol Psychiatry.

[B162] Drevets WC, Burton H, Videen TO, Snyder AZ, Simpson JR, Raichle ME (1995). Blood flow changes in human somatosensory cortex during anticipated stimulation. Nature.

[B163] Drevets WC, Price JL, Simpson JR, Todd RD, Reich T, Vannier M, Raichle ME (1997). Subgenual prefrontal cortex abnormalities in mood disorders. Nature.

[B164] Wu JC, Gillin JC, Buchsbaum MS, Hershey T, Johnson JC, Bunney WE (1992). Effect of sleep deprivation on brain metabolism of depressed patients. Am J Psychiatry.

[B165] Mentis MJ, Pietrini P, Polles A (1995). Cerebral glucose metabolism in late onset depression without cognitive impairment. Neurosci.

[B166] Abercrombie HC, Larson CL, Ward TL (1996). Metabolic rate in the amygdala predicts negative affect and depression severity in depressed patients: an FDG-PET study. Neuroimage.

[B167] Nofzinger EA, Nichols TE, Meltzer CC, Price J, Steppe DA, Miewald JM, Kupfer DJ, Moore RY (1999). Changes in forebrain function from waking to REM sleep in depression: preliminary analyses of [18F]FDG PET studies. Psychiatry Res.

[B168] Ketter TA, Kimbrell TA, George MS, Dunn RT, Speer AM, Benson BE, Willis MW, Danielson A, Frye MA, Herscovitch P, Post RM (2001). Effects of mood and subtype on cerebral glucose metabolism in treatment-resistant bipolar disorder. Biol Psychiatry.

[B169] Ketter TA, Wang PW (2002). Predictors of treatment response in bipolar disorders: evidence from clinical and brain imaging studies. J Clin Psychiatry.

[B170] Schneider F, Gur RE, Mozley LH, Smith RJ, Mozley PD, Censits DM, Alavi A, Gur RC (1995). Mood effects on limbic blood flow correlate with emotional self-rating: a PET study with oxygen-15 labeled water. Psychiatry Res.

[B171] Schneider F, Grodd W, Weiss U, Klose U, Mayer KR, Nägele T, Gur RC (1997). Functional MRI reveals left amygdala activation during emotion. Psychiatry Res.

[B172] Sheline YI, Barch DM, Donnelly JM, Ollinger JM, Snyder AZ, Mintun MA (2001). Increased amygdala response to masked emotional faces in depressed subjects resolves with antidepressant treatment: an fMRI study. Biol Psychiatry.

[B173] Fu CH, Williams SC, Cleare AJ, Brammer MJ, Walsh ND, Kim J, Andrew CM, Pich EM, Williams PM, Reed LJ, Mitterschiffthaler MT, Suckling J, Bullmore ET (2004). Attenuation of the neural response to sad faces in major depression by antidepressant treatment: a prospective, event-related functional magnetic resonance imaging study. Arch Gen Psychiatry.

[B174] Delgado JR, Hamlin H, Higgins JW, Mahl GF (1956). Behavioral changes during intracerebral electrical stimulation. AMA Arch Neurol Psychiatry.

[B175] Bickford RG, Mulder DW, Dodge HW, Svien HJ, Rome HP (1958). Changes in memory function produced by electrical stimulation of the temporal lobe in man. Res Pub Assoc Res Nerv Mental Dis.

[B176] Baldwin M, Ramey ER, Doherty DS (1960). Electrical stimulation of the mesial temporal region. Electrical studies on the unanesthetized brain.

[B177] Penfield W, Perrot P (1963). The brain's record of auditory and visual experience. Brain.

[B178] Horowitz MJ, Adams JE, Rutkin BB (1968). Visual imagery on brain stimulation. Arch Gen Psychiatry.

[B179] Ferguson SM, Rayport M, Gardner R, Kass W, Weiner H, Reiser MF (1969). Similarities in mental content of psychotic states, spontaneous seizures, dreams, and responses to electrical brain stimulation in patients with temporal lobe epilepsy. Psychosom Med.

[B180] Halgren E, Walter RD, Cherlow DG, Crandall PH (1978). Mental phenomena evoked by electrical stimulation of the human hippocampus formation and amygdala. Brain.

[B181] Wieser HG, ILAE Commission on Neurosurgery of Epilepsy (2004). ILAE Commission Report. Mesial temporal lobe epilepsy with hippocampal sclerosis. Epilepsia.

[B182] Bartolomei F, Barbeau E, Gavaret M, Guye M, McGonigal A, Regis J, Chauvel P (2004). Cortical stimulation study of the role of rhinal cortex in deja vu and reminiscence of memories. Neurology.

[B183] Bancaud J, Brunet-Bourgin F, Chauvel P, Halgren E (1994). Anatomical origin of déjà vu and vivid 'memories' in human temporal lobe epilepsy. Brain.

[B184] Barbeau E (2005). Recollection of vivid memories after perirhinal region stimulations: synchronization in the theta range of spatially distributed brain areas. Neuropsychologia.

[B185] Vignal JP, Maillard L, McGonigal A, Chauvel P (2007). The dreamy state: hallucinations of autobiographic memory evoked by temporal lobe stimulations and seizures. Brain.

[B186] Ostow M (1952). Psychodynamic disturbances in patients with temporal lobe disorders. Trans Am Neurol Assoc.

[B187] Ostow M (1954). Psychodynamic disturbances in patients with temporal lobe disorder. J Mt Sinai Hosp N Y.

[B188] Kubie LS (1953). Some implications for psychoanalysis of modern concepts of the organization of the brain. Psychoanal Q.

[B189] Rodin EA, Mulder DW, Faucett RL, Bickford RG (1955). Psychologic factors in convulsive disorders of focal origin. AMA Arch Neurol Psychiatry.

[B190] Epstein AW, Ervin F (1956). Psychodynamic significance of seizure content in psychomotor epilepsy. Psychosom Med.

[B191] Mahl GF, Rothenberg A, Delgado JMR, Hamlin H (1964). Psychological responses in the human to intracerebral electrical stimulation. Psychosom Med.

[B192] Ferguson SM, Rayport M (2006). Id, ego, and temporal lobe revisited. Int Rev Neurobiol.

[B193] Cohen S (1964). The Beyond Within The LSD Story.

[B194] Ongür D, An X, Price JL (1998). Prefrontal cortical projections to the hypothalamus in macaque monkeys. J Comp Neurol.

[B195] Freedman LJ, Insel TR, Smith Y (2000). Subcortical projections of area 25 (subgenual cortex) of the macaque monkey. J Comp Neurol.

[B196] Ongür D, An X, Price JL (2000). Prefrontal cortical projections to the hypothalamus in macaque monkeys. J Comp Neurol.

[B197] Kondo H, Saleem KS, Price JL (2005). Differential connections of the perirhinal and parahippocampal cortex with the orbital and medial prefrontal networks in macaque monkeys. J Comp Neurol.

[B198] Johansen-Berg H, Gutman DA, Behrens TE, Matthews PM, Rushworth MF, Katz E, Lozano AM, Mayberg HS (2008). Anatomical connectivity of the subgenual cingulate region targeted with deep brain stimulation for treatment-resistant depression. Cereb Cortex.

[B199] Lehéricy S, Ducros M, Moortele PF Van de, Francois C, Thivard L, Poupon C, Swindale N, Ugurbil K, Kim DS (2004). Diffusion tensor fiber tracking shows distinct corticostriatal circuits in humans. Ann Neurol.

[B200] Palomero-Gallagher N, Mohlberg H, Zilles K, Vogt B (2008). Cytology and receptor architecture of human anterior cingulate cortex. J Comp Neurol.

[B201] Pezawas L, Meyer-Lindenberg A, Drabant EM, Verchinski BA, Munoz KE, Kolachana BS, Egan MF, Mattay VS, Hariri AR, Weinberger DR (2005). 5-HTTLPR polymorphism impacts human cingulate-amygdala interactions: a genetic susceptibility mechanism for depression. Nat Neurosci.

[B202] Haas BW, Omura K, Constable RT, Canli T (2007). Emotional conflict and neuroticism: personality-dependent activation in the amygdala and subgenual anterior cingulate. Behav Neurosci.

[B203] Mogenson GJ, Swanson LW, Wu M (1983). Neural projections from nucleus accumbens to globus pallidus, substantia innominata, and lateral preoptic-lateral hypothalamic area: an anatomical and electrophysiological investigation in the rat. J Neurosci.

[B204] Di Martino A, Scheres A, Margulies DS, Kelly AM, Uddin LQ, Shehzad Z, Biswal B, Walters JR, Castellanos FX, Milham MP (2008). Functional connectivity of human striatum: a resting state fMRI study. Cereb Cortex.

[B205] Leichnetz GR, Astruc J (1976). The efferent projections of the medial prefrontal cortex in the squirrel monkey (*Saimiri sciureus*). Brain Res.

[B206] Heath RG (1963). Electrical stimulation of the brain in man. Am J Psychiatry.

[B207] Heath RG (1972). Pleasure and brain activity in man. Deep and surface electroencephalograms during orgasm. J Nerv Mental Dis.

[B208] Schlaepfer TE, Cohen MX, Frick C, Kosel M, Brodesser D, Axmacher N, Joe AY, Kreft M, Lenartz D, Sturm V (2008). Deep brain stimulation to reward circuitry alleviates anhedonia in refractory major depression. Neuropsychopharmacology.

[B209] Uranova NA, Zimina IS, Vikhreva OV, Denisov DV, Orlovskaya DD (1999). Morphometric study of ultrastructural alterations of myelinated fibres in post-mortem schizophrenia brains. Schizophr Res.

[B210] Johnston-Wilson NL, Sims CD, Hofmann JP, Anderson L, Shore AD, Torrey EF, Yolken RH (2000). Disease-specific alterations in frontal cortex brain proteins in schizophrenia, bipolar disorder, and major depressive disorder. The Stanley Neuropathology Consortium. Mol Psychiatry.

[B211] Rajkowska G (2000). Postmortem studies in mood disorders indicate altered numbers of neurons and glial cells. Biol Psychiatry.

[B212] Rajkowska G, Miguel-Hidalgo JJ, Wei J, Dilley G, Pittman SD, Meltzer HY, Overholser JC, Roth BL, Stockmeier CA (1999). Morphometric evidence for neuronal and glial prefrontal cell pathology in major depression. Biol Psychiatry.

[B213] Bremner JD, Vythilingam M, Vermetten E, Nazeer A, Adil J, Khan S, Staib LH, Charney DS (2002). Reduced volume of orbitofrontal cortex in major depression. Biol Psychiatry.

[B214] Cotter D, Mackay D, Chana G, Beasley C, Landau S, Everall IP (2002). Reduced neuronal size and glial cell density in area 9 of the dorsolateral prefrontal cortex in subjects with major depressive disorder. Cereb Cortex.

[B215] Benes FM, Davidson J, Bird ED (1986). Quantitative cytoarchitectural studies of the cerebral cortex of schizophrenics. Arch Gen Psychiatry.

[B216] Benes FM, McSparren J, Bird ED, San Giovanni JP, Vincent SL (1991). Deficits in small interneurons in prefrontal and cingulate cortices of schizophrenic and schizoaffective patients. Arch Gen Psychiatry.

[B217] Ongür D, Drevets WC, Price JL (1998). Glial reduction in the subgenual prefrontal cortex in mood disorders. Proc Natl Acad Sci USA.

[B218] Orlovskaya DD, Vostrikov VM, Rachmanova VI, Uranova NA (2000). Decreased numerical density of oligodendroglial density cells in the prefrontal cortex area 9 in schizophrenia and mood disorders: a study of brain collection from the Stanley Foundation Neuropathology Consortium. Schizophr Res.

[B219] Cotter D, Mackay D, Landau S, Kerwin R, Everall I (2001). Reduced glial cell density and neuronal size in the anterior cingulate cortex in major depressive disorder. Arch Gen Psychiatry.

[B220] Hirayasu Y, Shenton ME, Salisbury DF, Kwon JS, Wible CG, Fischer IA, Yurgelun-Todd D, Zarate C, Kikinis R, Jolesz FA, McCarley RW (1999). Subgenual cingulate cortex volume in first-episode psychosis. Am J Psychiatry.

[B221] Botteron KN, Raichle ME, Drevets WC, Heath AC, Todd RD (2002). Volumetric reduction in left subgenual prefrontal cortex in early onset depression. Biol Psychiatry.

[B222] MacQueen GM, Campbell S, McEwen BS, Macdonald K, Amano S, Joffe RT, Nahmias C, Young LT (2003). Course of illness, hippocampal function, and hippocampal volume in major depression. Proc Natl Acad Sci USA.

[B223] Sheline YI, Wang PW, Gado MH, Csernansky JG, Vannier MW (1996). Hippocampal atrophy in recurrent major depression. Proc Natl Acad Sci USA.

[B224] Sheline YI, Sanghavi M, Mintun MA, Gado MH (1999). Depression duration but not age predicts hippocampal volume loss in medically healthy women with recurrent major depression. J Neurosci.

[B225] Bell-McGinty S, Butters MA, Meltzer CC, Greer PJ, Reynolds CF, Becker JT (2002). Brain morphometric abnormalities in geriatric depression: long-term neurobiological effects of illness duration. Am J Psychiatry.

[B226] Steffens DC, Byrum CE, McQuoid DR, Greenberg DL, Payne ME, Blitchington TF, MacFall JR, Krishnan KR (2003). Hippocampal volume in geriatric depression. Biol Psychiatry.

[B227] Mervaala E, Föhr J, Könönen M, Valkonen-Korhonen M, Vainio P, Partanen K, Partanen J, Tiihonen J, Viinamäki H, Karjalainen AK, Lehtonen J (2000). Quantitative MRI of the hippocampus and amygdala in severe depression. Psychol Med.

[B228] Bremner JD, Narayan M, Anderson ER, Staib LH, Miller HL, Charney DS (2000). Hippocampal volume reduction in major depression. Am J Psychiatry.

[B229] Shah PJ, Ebmeier KP, Glabus MF, Goodwin GM (1998). Cortical grey matter reductions associated with treatment-resistant chronic unipolar depression. Controlled magnetic resonance imaging study. Br J Psychiatry.

[B230] Pearlson GD, Barta PE, Powers RE, Menon RR, Richards SS, Aylward EH, Federman EB, Chase GA, Petty RG, Tien AY (1997). Medial and superior temporal gyral volumes and cerebral asymmetry in schizophrenia versus bipolar disorder. Biol Psychiatry.

[B231] Bowley MP, Drevets WC, Ongür D, Price JL (2002). Low glial numbers in the amygdala in major depressive disorder. Biol Psychiatry.

[B232] Frodl T, Meisenzahl EM, Zetzsche T, Born C, Groll C, Jäger M, Leinsinger G, Bottlender R, Hahn K, Möller HJ (2002). Hippocampal changes in patients with a first episode of major depression. Am J Psychiatry.

[B233] Baumann B, Danos P, Krell D, Diekmann S, Leschinger A, Stauch R, Wurthmann C, Bernstein HG, Bogerts B (1999). Reduced volume of limbic system-affiliated basal ganglia in mood disorders: preliminary data from a postmortem study. J Neuropsychiatry Clin Neurosci.

[B234] Krishnan KR, McDonald WM, Escalona PR, Doraiswamy PM, Na C, Husain MM, Figiel GS, Boyko OB, Ellinwood EH, Nemeroff CB (1992). Magnetic resonance imaging of the caudate nuclei in depression. Preliminary observations. Arch Gen Psychiatry.

[B235] Sapolsky RM (2000). Glucocorticoids and hippocampal atrophy in neuropsychiatric disorders. Arch Gen Psychiatry.

[B236] Nemeroff CB (1996). The corticotropin-releasing factor (CRF) hypothesis of depression: new findings and new directions. Mol Psychiatry.

[B237] Drevets WC (2003). Neuroimaging abnormalities in the amygdala in mood disorders. Ann NY Acad Sci.

[B238] Rubin RT, Mandell AJ, Crandall PH (1966). Corticosteroid responses to limbic stimulation in man: localization of stimulus sites. Science.

[B239] Sheline YI (2003). Neuroimaging studies of mood disorder effects on the brain. Biol Psychiatry.

[B240] Finch DM, Vogt BA, Gabriel M (1993). Hippocampal, subicular, and entorhinal afferents and synaptic integration in rodent cingulate cortex. Neurobiology of cingulate cortex and the limbic thalamus: a comprehensive handbook.

[B241] Falkai P, Bogerts B (1986). Cell loss in the hippocampus of schizophrenics. Eur Arch Psychiatry Neurol Sci.

[B242] Falkai P, Bogerts B, Rozumek M (1988). Limbic pathology in schizophrenia: the entorhinal region – a morphometric study. Biol Psychiatry.

[B243] Bogerts B, Häntsch J, Herzer M (1983). A morphometric study of the dopamine-containing cell groups in the mesencephalon of normals, Parkinson patients, and schizophrenics. Biol Psychiatry.

[B244] Radewicz K, Garey LJ, Gentleman SM, Reynolds R (2000). Increase in HLA-DR immunoreactive microglia in frontal and temporal cortex of chronic schizophrenics. J Neuropathol Exp Neurol.

[B245] Orlovskaya DD, Vikhreva OV, Zimina IS, Denisov DV, Uranova NA (1999). Ultrastructural dystrophic changes of oligodendroglial cells in autopsied prefrontal cortex and striatum in schizophrenia: a morphometric study. Schizophr Res.

[B246] Webster MJ, Johnston-Wilson N, Nagata K, Yolken RH (2000). Alterations in the expression of phosphorylated glial fibrillary acidic proteins in the frontal cortex of individuals with schizophrenia, bipolar disorder, and depression. Schizophr Res.

[B247] Honer WG, Falkai P, Chen C, Arango V, Mann JJ, Dwork AJ (1999). Synaptic and plasticity-associated proteins in anterior frontal cortex in severe mental illness. Neurosci.

[B248] Owen F, Crow TJ, Frith CD, Johnson JA, Johnstone EC, Lofthouse R, Owens DG, Poulter M (1987). Selective decreases in MAO-B activity in post-mortem brains from schizophrenic patients with type II syndrome. Br J Psychiatry.

[B249] Rubin E, Sackeim HA, Prohovnik I, Moeller JR, Schnur DB, Mukherjee S (1995). Regional cerebral blood flow in mood disorders: IV. Comparison of mania and depression. Psychiatry Res.

[B250] Blumberg HP, Stern E, Martinez D, Ricketts S, de Asis J, White T, Epstein J, McBride PA, Eidelberg D, Kocsis JH, Silbersweig DA (2000). Increased anterior cingulate and caudate activity in bipolar mania. Biol Psychiatry.

[B251] Goodwin GM, Cavanagh JT, Glabus MF, Kehoe RF, O'Carroll RE, Ebmeier KP (1997). Uptake of 99mTc-exametazime shown by single photon emission computed tomography before and after lithium withdrawal in bipolar patients: associations with mania. Br J Psychiatry.

[B252] Freud S (1926). Inhibitions, symptoms and anxiety.

[B253] Neimat JS, Hamani C, Giacobbe P, Merskey H, Kennedy SH, Mayberg HS, Lozano AM (2008). Neural stimulation successfully treats depression in patients with prior ablative cingulotomy. Am J Psychiatry.

[B254] McNeely HE, Mayberg HS, Lozano AM, Kennedy SH (2008). Neuropsychological impact of Cg25 deep brain stimulation for treatment-resistant depression: preliminary results over 12 months. J Nerv Mental Dis.

[B255] Eslinger PJ, Damasio AR (1985). Severe disturbance of higher cognition after bilateral frontal lobe ablation: patient EVR. Neurology.

[B256] Cummings JL (1985). Clinical neuropsychiatry.

[B257] Beer JS, Heerey EA, Keltner D, Scabini D, Knight RT (2003). The regulatory function of self-conscious emotion: insights from patients with orbitofrontal damage. J Personal Soc Psychol.

[B258] Moretti L, Dragone D, di Pellegrino G (2008). Reward and social valuation deficits following ventromedial prefrontal damage. J Cogn Neurosci.

[B259] Seeley WW, Menon V, Schatzberg AF, Keller J, Glover GH, Kenna H, Reiss AL, Greicius MD (2007). Dissociable intrinsic connectivity networks for salience processing and executive control. J Neurosci.

[B260] Goethe JWV (1808). Faust The first part of the tragedy.

[B261] Albus JS, Bekey GA, Holland JH, Kanwisher NG, Krichmar JL, Mishkin M, Modha DS, Raichle ME, Shepherd GM, Tononi GA (2007). Proposal for a Decade of the Mind initiative. Science.

[B262] Sacks O (1984). A leg to stand on.

[B263] Kandel ER (1999). Biology and the future of psychoanalysis: a new intellectual framework for psychiatry revisited. Am J Psychiatry.

[B264] Hering E (1870). Uber das Gedachtnis al seine allgemaine Function der organisirten Materie.

[B265] Freud S (1925). An autobiographical study.

[B266] Abramson HA (1967). The second international conference on the use of LSD in psychotherapy.

[B267] Martin AJ (1962). The treatment of twelve male homosexuals with LSD. Acta Psychother.

[B268] Grof S (1975). Realms of the human unconscious Observations from LSD research.

[B269] Sandison RA (1954). Psychological aspects of the LSD treatment of the neuroses. J Ment Sci.

[B270] Sandison RA (1957). The contribution of LSD therapy to analytic theory and practice. Bull Br Psychol Soc.

[B271] Sandison RA, Crocket R, Sandison RA, Walk A (1963). Certainty and uncertainty in the LSD treatment of psychoneurosis. Hallucinogenic drugs and their psychotherapeutic use.

[B272] Lewis DJ, Sloane RB (1958). Therapy with lysergic acid diethylamide. J Clin Exp Psychopathol.

[B273] Cutner M (1959). Analytic work with LSD 25. Psychiatr Q.

[B274] Rolo A, Krinsky LW, Goldfarb L (1960). LSD as an adjunct to psychotherapy with alcoholics. J Psychol.

[B275] Ling TM, Abramson HA (1967). The use of LSD and ritalin in the treatment of neurosis. The second international conference on the use of LSD in psychotherapy.

[B276] Spencer AM (1963). Permissive group therapy with lysergic acid diethylamide. Br J Psychiatry.

[B277] Spencer AM (1964). Modifications in the technique of LSD therapy. Comp Psychiatry.

[B278] Abramson HA (1956). Lysergic acid diethylamide (LSD-25): XIX. As an adjunction to brief psychotherapy with special reference to ego enhancement. J Psychology.

[B279] Abramson HA (1959). The use of LSD in psychotherapy.

[B280] Leuner H, Abramson HA (1967). Present state of psycholytic therapy and its possibilities. The second international conference on the use of LSD in psychotherapy.

[B281] Osmond H, Abramson HA (1967). A comment on some uses of psychotomimetics in psychiatry. The second international conference on the use of LSD in psychotherapy.

[B282] Busch AK, Johnson WC (1950). LSD-25 as an aid to psychotherapy. Disease Nerv Sys.

[B283] Grof S, Abramson HA (1967). The use of LSD-25 in personality diagnostics and therapy of psychogenic disorders. The second international conference on the use of LSD in psychotherapy.

[B284] Grof S (1980). LSD psychotherapy.

[B285] Eisner BG (1959). Communication to first international congress of CNIP, Rome. The use of LSD in psychotherapy.

[B286] Vollenweider FX, Leenders KL, Scharfetter C, Maguire P, Stadelmann O, Angst J (1997). Positron emission tomography and fluorodeoxyglucose studies of metabolic hyperfrontality and psychopathology in the psilocybin model of psychosis. Neuropsychopharmacology.

[B287] Hermle L, Fünfgeld M, Oepen G, Botsch H, Borchardt D, Gouzoulis E, Fehrenbach RA, Spitzer M (1992). Mescaline-induced psychopathological, neuropsychological, and neurometabolic effects in normal subjects: experimental psychosis as a tool for psychiatric research. Biol Psychiatry.

[B288] Freud S (1923). Two encyclopaedia articles.

[B289] Buckner RL, Andrews-Hanna JR, Schacter DL (2008). The brain's default network: anatomy, function, and relevance to disease. Ann NY Acad Sci.

